# Gaptronics: multilevel photonics applications spanning zero-nanometer limits

**DOI:** 10.1515/nanoph-2021-0798

**Published:** 2022-03-24

**Authors:** Jeeyoon Jeong, Hyun Woo Kim, Dai-Sik Kim

**Affiliations:** Department of Physics and Institute of Quantum Convergence Technology, Kangwon National University, Chuncheon, Gangwon 24341, Korea; Laboratory for Advanced Molecular Probing (LAMP), Korea Research Institute of Chemical Technology, Daejeon 34114, Korea; Department of Physics and Astronomy, Seoul National University, Seoul 08826, Korea; Department of Physics and Center for Atom Scale Electromagnetism, Ulsan National Institute of Science and Technology (UNIST), Ulsan 44919, Korea; Quantum Photonics Institute, Ulsan National Institute of Science and Technology (UNIST), Ulsan 44919, Korea

**Keywords:** light–matter interaction, lithography, nanophotonics, reconfigurable metasurface, sub-nanometer, wafer-scale, zerogap

## Abstract

With recent advances in nanofabrication technology, various metallic gap structures with gap widths reaching a few to sub-nanometer, and even ‘zero-nanometer’, have been realized. At such regime, metallic gaps not only exhibit strong electromagnetic field confinement and enhancement, but also incorporate various quantum phenomena in a macroscopic scale, finding applications in ultrasensitive detection using nanosystems, enhancement of light–matter interactions in low-dimensional materials, and ultralow-power manipulation of electromagnetic waves, etc. Therefore, moving beyond nanometer to ‘zero-nanometer’ can greatly diversify applications of metallic gaps and may open the field of dynamic ‘gaptronics.’ In this paper, an overview is given on wafer-scale metallic gap structures down to zero-nanometer gap width limit. Theoretical description of metallic gaps from sub-10 to zero-nanometer limit, various wafer-scale fabrication methods and their applications are presented. With such versatility and broadband applicability spanning visible to terahertz and even microwaves, the field of ‘gaptronics’ can be a central building block for photochemistry, quantum optical devices, and 5/6G communications.

## 1 Electromagnetic description of metallic gaps down to zero-nanometer limit

Subwavelength openings in metals can create unusual electromagnetic environments given the right geometry [1, 2]. Unlike in circular subwavelength holes easily circumvented by the eddy currents driven by the magnetic field of light, long and narrow slots facing the eddy current in the direction of $\hat{n}\times \overset{\to }{H}$ ( $\hat{n}$ = surface normal; $\vec{H}$ = magnetic field) are subject to capacitive accumulation of surface charges resulting in enhanced electric field at the narrow gap. Often accompanied by plasmonic and/or geometric resonances, these giant field enhancements lie at the heart of currently flourishing field of nanophotonics [3, 4]. While the word ‘nano’ seems to prefer shorter wavelengths, in truth the principle of nanophotonics is ubiquitously used throughout ultraviolet, visible, infrared, and even up to millimeter wavelengths and microwaves. The field enhancementis important in the context of controlling propagation of light in the vector Kirchhoff diffraction formalism sense, but also plays crucial roles in modulating light–matter interactions, which has led to numerous applications including sensing [5], [6], [7], [8], [9], [10], [11], [12], nonlinear optics [13], [14], [15], [16], [17], [18], strong light–matter interaction [19], [20], [21], [22], quantum optics [23, 24], etc.

**Figure 1:**
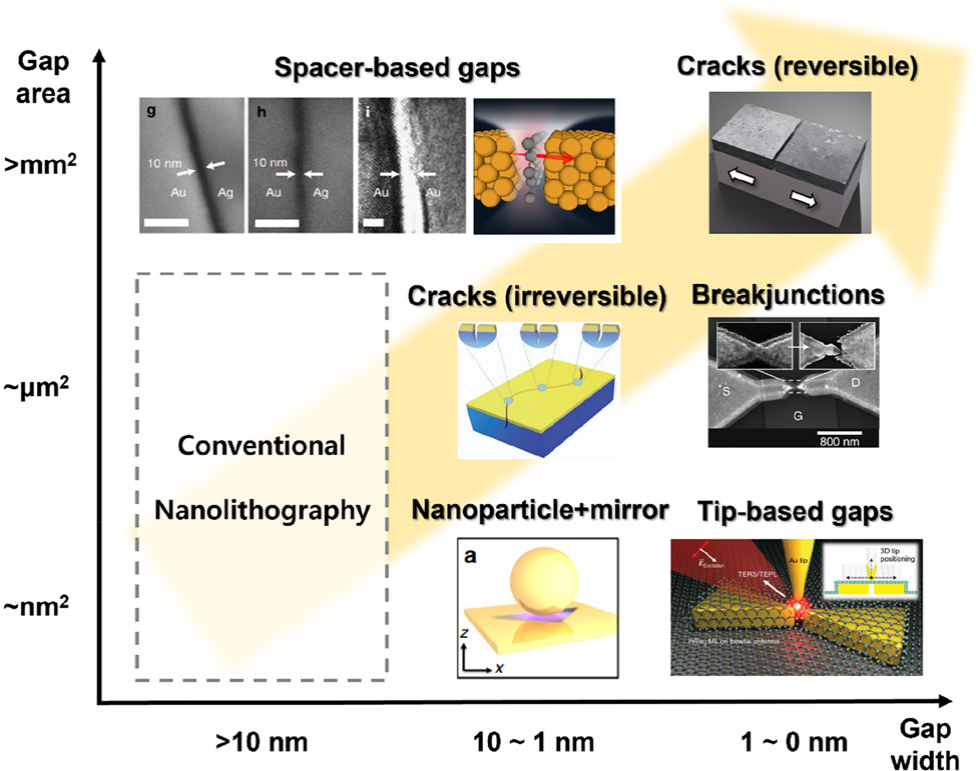
An overview on various types of metallic gaps depending on minimum achievable gap widths and capability of fabrication over a large area. Reproduced with permission from [25, 26] (Spacer-based gaps) [27], (cracks – reversible) [28], (cracks-irreversible) [29], (breakjunctions) [30], (nanoparticle + mirror) [31], (tip-based gaps).

As a smaller gap generally leads to a stronger field enhancement owing to the shorter distances between the oscillating plus and minus charges at the opposing surfaces, much effort has been made to reduce the gap size. While the state-of-the-art standard lithographic techniques show resolution of ∼10 nm or slightly less, for certain types of structures gap width of ∼1 nm could be achieved in wafer-scale with essentially no limitations in operating wavelengths. There are also recent reports on subnanometer-wide and even ‘zero-nanometer’ gaps, in which case local approximation breaks down. For such structures classical Maxwell equations with the assumption of infinitely sharp interfaces are not enough to describe their optical properties and quantum mechanical correction is required. For extended gap geometries such as slits and slots, which are frequently used in long wavelength applications, occasional formation of conducting channels within the gap must also be considered at sub- to zero-nanometer regimes. Aforementioned emerging phenomena greatly extend the applicability of gap structures beyond the simple picture of electromagnetic field enhancement. When combined with wafer-scale fabrication capability, metallic nanogaps can be a central building block for future integrated optical and optoelectronic devices, and we call this burgeoning research area ‘gaptronics’ (Figure 1).

In this chapter, we discuss electromagnetic description of metallic gap structures in three distinct regimes of the gap width: (1) sub-skin-depth, (2) few- to sub-nanometer, and (3) ‘zero-nanometer’. Each regime is associated with different classes of assumptions on the properties of metallic gaps – large but finite permittivity of metal, conducting electrons with nonlocal response, and non-uniform surfaces leading to conducting channels, etc. Detailed shape of the gap – point-like or extended – also matters. The following sections will give a brief overview on theoretical backgrounds and insights into the observed behaviors of such gap structures.

### 1.1 Sub-skin-depth-wide gap: gap plasmon modes

**Figure 2:**
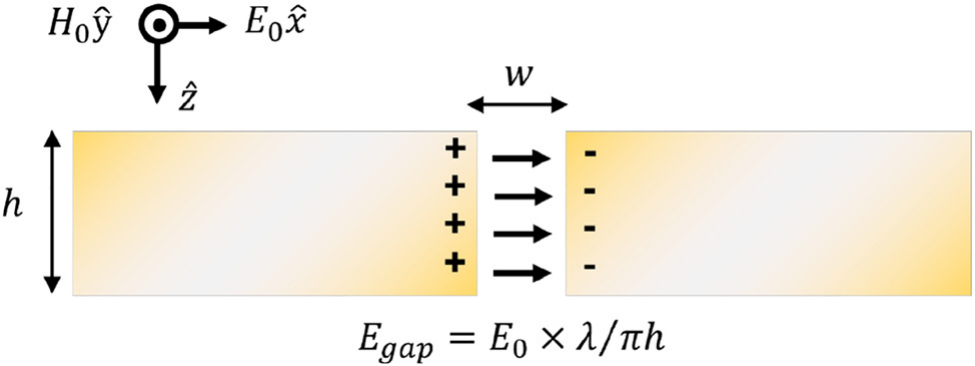
Cross-sectional schematic sketch of an infinite slit with width *w* and thickness *h*.

A gap structure made of an infinitely thin perfect electric conductor (PEC) can incorporate infinitely large electric field enhancement at the limit of zero-nanometer gap width. In practice this does not happen because of finite film thickness, finite electric permittivity; conductivity, of metals, and there exists a limit where the electric field enhancement saturates as the gap width decreases [32, 33]. This is true even for low frequency regimes such as terahertz (THz) or microwave frequencies where permittivity of Drude metals diverge as ∼1/ω and reaches $10^{6}$ or higher. Yet, due to difficulties in simulating nanometric structures operating at millimeter or longer wavelengths, properties of nanogaps appear not well understood in this wavelength range compared to those at visible or near-infrared. This situation can be paradoxically simplified for an infinitely long, ultranarrow slits at long wavelengths (Figure 2), which gives rise to the well-known formula of the electric field enhancement independent of the gap width [34],

(1) $$\frac{E_{\text{gap}}}{E_{0}}=\frac{\lambda }{\pi h},$$

where $E_{0}$ is the incident electric field, $E_{\text{gap}}$ is the gap electric field, λ is the electromagnetic wavelength, and h is thickness of the metal film. A heuristic derivation of Eq. (1), which is applicable in the limit of h≫w , can be informative here. With the assumption of small transmission, large reflection, and metal thickness of the order the skin depth or less, the current density J inside the conductor is given by the Ampere’s law $Jh=2H_{0}$ whereby the total surface current density K=Jh blocks transmission through destructive interference. With the continuity equation J=σω (*σ*: surface charge density at the facets) and Gauss’s law, we arrive at

(2) $$E_{\text{gap}}=\frac{\sigma }{\epsilon_{0}}=\frac{J}{\omega \epsilon_{0}}=\frac{2H_{0}}{\omega \epsilon_{0}h}=\frac{\lambda }{\pi h}\frac{H_{0}}{\epsilon_{0}c}=\frac{\lambda }{\pi h}E_{0}.$$

For an array of slits, Fabry–Perot effects may lead to plasmonic band gap structure and gap can incorporate much higher $E_{\text{gap}}$ [35, 36]. Also, we briefly comment that this enhancement factor decreases by a factor $n_{\text{eff}}=\sqrt{\left(n_{1}^{2}+n_{3}^{2}\right)/2}$ when media with refractive indices of $n_{1}$ and $n_{3}$ are filled above and below the gap, respectively (i.e., in the presence of a substrate) [37], [38], [39]. In this subsection we will mainly discuss the effect of very large but finite permittivity of metals in the long wavelength regime on optical properties of gap structures.

For a prototypical metallic gap structure of a rectangular hole, effects associated with having a real metal instead of a perfect electric conductor can be addressed by adding surface impedance boundary conditions and by accounting for an effective index of the gap [40, 41]. For long wavelength radiations, permittivity of noble metals is very high and corrections from surface impedances are very small (less than 1 percent for THz frequencies). Most changes in optical properties therefore come from the gap plasmon effect, which are essentially two coupled surface plasmon modes in a metal–insulator–metal (MIM) structure [39]. At the lowest order gap plasmon mode, *x*-components of the electric fields in the gap and the metal have the form

(3) $$E_{x,\text{gap}}\propto {\text{e}}^{i\beta z}\;\text{cosh}\;k_{\text{gap}}x,\left|x\right|<w/2,$$

(4) $$E_{x,\text{metal}}\propto {\text{e}}^{i\beta z}{\text{e}}^{-k_{\text{metal}}\left(\left|x\right|-w/2\right)},\left|x\right|>w/2,$$

assuming that the two metallic layers are identical. Here, β is the propagation constant of the gap plasmon mode, w is the gap width, and $k_{\text{gap}}$ $\left(k_{\text{metal}}\right)$ denotes wavevector component perpendicular to the interface in the gap (metal). As we will briefly see, at sub-skin-depth gap widths $k_{\text{gap}}$ becomes much larger than $k_{0}$ and $\left|k_{\text{gap}}\right|≃\left|\beta \right|$ . Solving for boundary conditions and the wave equation leads to the following dispersion relation,

(5) $$\text{tanh}\left(k_{\text{gap}}w/2\right)=-k_{\text{metal}}\epsilon_{\text{gap}}/k_{\text{gap}}\epsilon_{\text{metal}}.$$

where $\epsilon_{\text{gap}}$ $\left(\epsilon_{\text{metal}}\right)$ are electric permittivity of the gap material (metal). In contrast to surface plasmon dispersion relation in a single metal–insulator interface, the dispersion relation for gap plasmons has no cutoff frequency such that long wavelength radiations such as millimeter waves and microwaves can also be affected by the gap plasmon effect. Especially, at such long wavelength limit, electric permittivity of metal becomes orders of magnitude larger than any other permittivity of our interest and the relation is simplified to

(6) $$k_{\text{gap}}^{2}/k_{0}^{2}≃i\delta /w,\epsilon_{\text{metal}}\gg 1.$$

Here, $k_{0}=2\pi /\lambda$ is the vacuum wavevector, $\delta =\sqrt{2/\sigma \omega \mu_{0}}$ is the skin depth of metal at an angular frequency $\omega =ck_{0}$ and σ is the DC conductivity of the metal. Equation (6) implies that the gap plasmon modes start to dominate original waveguide mode and alter the effective index inside the gap when the gap width becomes smaller than the skin depth of metal. The skin depth of bulk gold at a frequency of 1 THz is ∼140 nm, which is three orders of magnitude smaller than the wavelength of interest but is much larger than the visible wavelengths. This means that for the same gap width the effect of gap plasmon can be much more pronounced in structures resonant at longer wavelengths, such that the observed resonant frequencies and transmission/reflection deviate significantly from the expected values when metals are perfect electric conductors [42]. Note that the required gap width is much smaller than the resolution of photolithography, while standard nano-lithographic tools are not well suited for fabricating millimeter-long structures with nanometric widths. Means to experimentally realize such structures will be discussed in Chapter 2.

**Figure 3:**
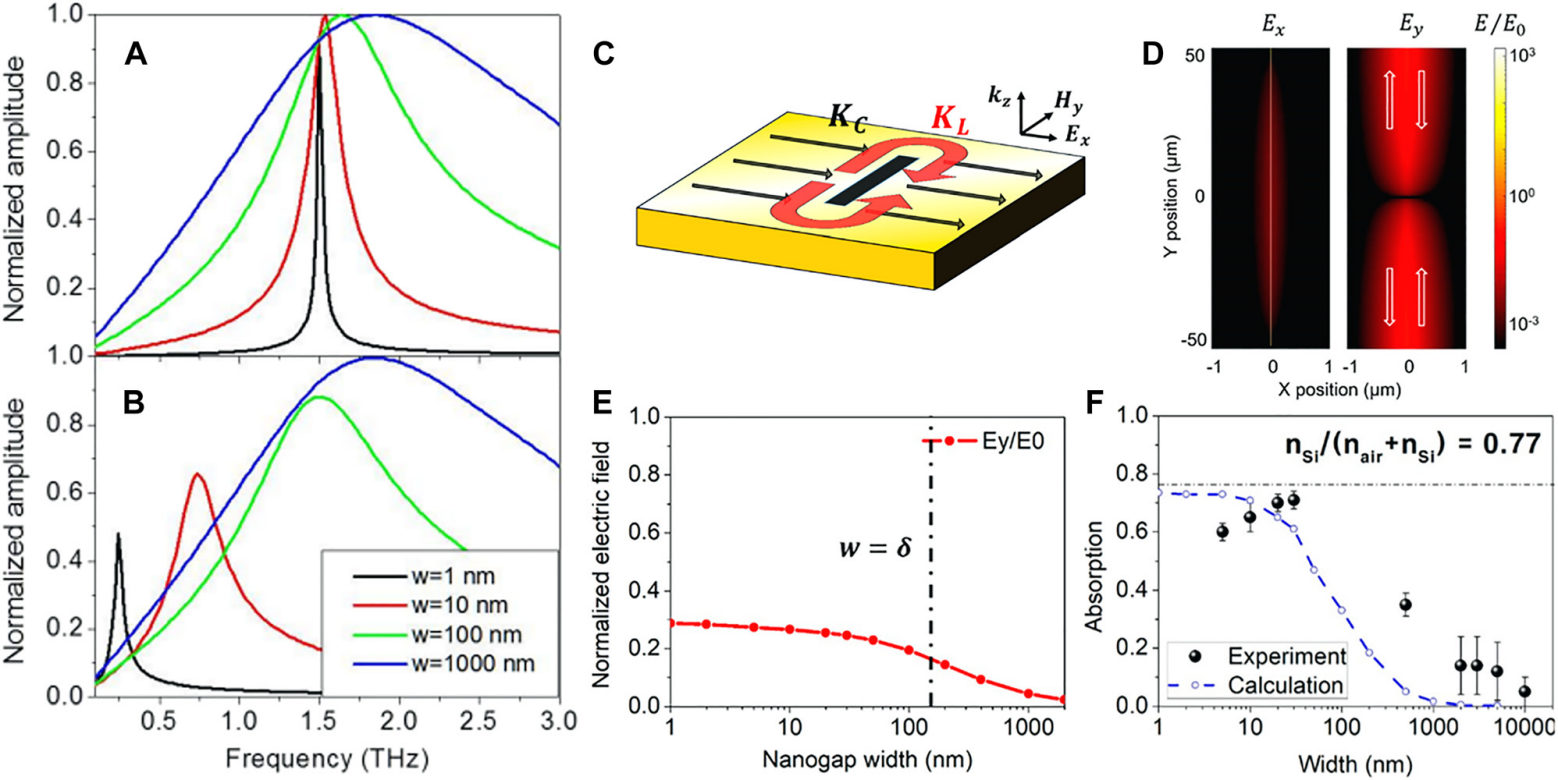
Gap plasmon effects in sub-skin-depth-wide metallic nanogaps. (A) and (B) Calculated transmission spectra of metallic nanogaps with different widths *w*, assuming A perfect conductor and B gold as the metal, respectively. (C) Two current components that contribute to electric field enhancement in nanoslot antennas. Despite commonly accepted picture of capacitive field enhancement at the gap, inductive current *K*_L_ can be the dominant contribution over its capacitive counterpart *K*_C_. (D) Calculated electric field in the vicinity of a 10 nm-wide metallic gap. (E) Maximum amplitude of the inductive field *E*_*y*_ as a function of gap width. Dashed line denotes skin depth *δ* at 1 THz. F Measured and calculated peak absorption of terahertz radiation in nanoslots with different widths. Reproduced with permission from [42] (A) and (B) and [43] (C)–(F).

Figure 3A and B show simulated transmission spectra of metallic nanoslots operating at terahertz frequencies, assuming perfect electric conductor and gold, respectively. The effect of real metal starts to appear at gap widths smaller than 100 nm (note that this value is very similar to the skin depth of gold at terahertz frequencies), leading to a decreased transmission and a red-shifted resonance due to gap plasmon-induced increase in effective indices of the gap. For slots with 1 nm gap width, calculations assuming real metal show a two-fold decrease in amplitude and six-fold decrease in resonance frequency. Owing to such drastic changes in optical properties of the gap, nanophotonic systems operating at long wavelengths can be an ideal testbed for quantitative analysis on light–matter interactions in nanoscale; millimeter and centimeter waves and nanostructures can be good bedfellows.

Also, as the word ‘plasmon’ in gap plasmon mode implies, significant Ohmic absorption can take place in metallic sub-skin depth gap structures operating at millimeter wavelengths even though noble metals at such long wavelengths are usually considered nearly perfect conductors. While there are several reports on large Ohmic absorption of long wavelength radiations in metallic metasurfaces [44, 45], absorption in the metallic gap structure is unique because it only takes place within the skin depth distance from the gap [43]. This is because the current (or equivalently, electric field inside metal $\vec{E}=\sigma^{-1}\vec{J}$ ) that drives the electric field enhancement in the gap is strongly localized to the vicinity of the gap. In a prototypical example of a nanoslot, as depicted in Figure 3C, the main driving field is inductive (flowing parallel to the long axis) and converges to $∼\left(\pi w/2l\right)E_{\text{gap}}$ as the gap width becomes much smaller than the skin depth, where $E_{\text{gap}}$ denotes the enhanced electric field inside the gap. For ∼10 nm-wide slot antennas the inductive field inside metal becomes as large as ∼30 percent of the incident field, which is ∼2 orders of magnitude larger than the capacitive counterpart (which flows perpendicular to the long axis of the slot) which scales as $∼\sigma \left(\epsilon_{\text{gap}}/\epsilon_{\text{metal}}\right)E_{\text{gap}}$ due to the giant permittivity contrast at the metal–dielectric boundary. This leads to ∼10^4^-fold enhancement in absorption density $P=\sigma E^{2}$ such that more than 70 percent of absorption takes place within 1% of the unit cell area.

### 1.2 Few to sub-nanometer gap: breakdown of local response approximation

When describing the relation between electric field ***E*** and displacement field ***D*** within a metal, a familiar expression would read

(7) $$D\left(r,\omega \right)=\epsilon_{0}\epsilon \left(\omega \right)E\left(r,\omega \right),$$

where $\epsilon_{0}$ is the vacuum permittivity, and ϵ(ω) denotes the metal permittivity generally described by the Drude-like dielectric function $\epsilon \left(\omega \right)=\epsilon_{\infty }-\omega_{\text{p}}^{2}/\omega \left(\omega +i\gamma \right)$ . This relation assumes that the response of electrons to the incident electric field is localized, which is usually referred to as local response approximation (LSA) [46]. In general, LSA is sufficient to describe most macroscopic phenomena and nanoplasmonic systems including nanoparticles, waveguides, metallodielectric multilayers, and hole arrays [47], [48], [49], [50], [51].

When the size of the plasmonic system decreases down to a few nanometers or less, nonlocal optical response of electrons needs to be considered. Nonlocality implies that the permittivity of metal at a certain position can affect the displacement field at *another* place. The equation above is therefore generalized to the following constitutive relation,

(8) $$D\left(r,\omega \right)=\epsilon_{0}\int \text{d}r^{\prime }\epsilon \left(r,r^{’},\omega \right)E\left(\mathrm{r’},\omega \right).$$

If we assume that the medium (here, metal) is homogeneous, the nonlocal permittivity $\epsilon \left(r,r^{’},\omega \right)=\epsilon \left(r-r^{’},\omega \right)$ and the equation now becomes a convolution integral, leading to the following relation,

(9) $$D\left(k,\omega \right)=\epsilon_{\text{o}}\epsilon \left(k,\omega \right)E\left(k,\omega \right).$$

The *k*-dependent permittivity can be theoretically addressed using *ab initio* calculations such as density functional theory [52], [53], [54]. Due to computational limits and for the sake of physical insights, however, hydrodynamic models are widely being used to describe the effect of nonlocal electronic response [55]. In such models, additional convection and diffusion currents appear as pressure terms that will act to balance inhomogeneity in the electron density. Such nonlocal effects are known to affect linewidth broadening or resonance shifts of surface plasmon modes in nanoparticles with different sizes [56, 57], and in multipole plasmons on a simple metal–dielectric interface [58], etc. For a metallic gap structure, the nonlocal effect manifest itself as broadening of the effective gap width or as an additional charge transfer mode, depending on whether the gap width reaches subnanometer or not.

**Figure 4:**
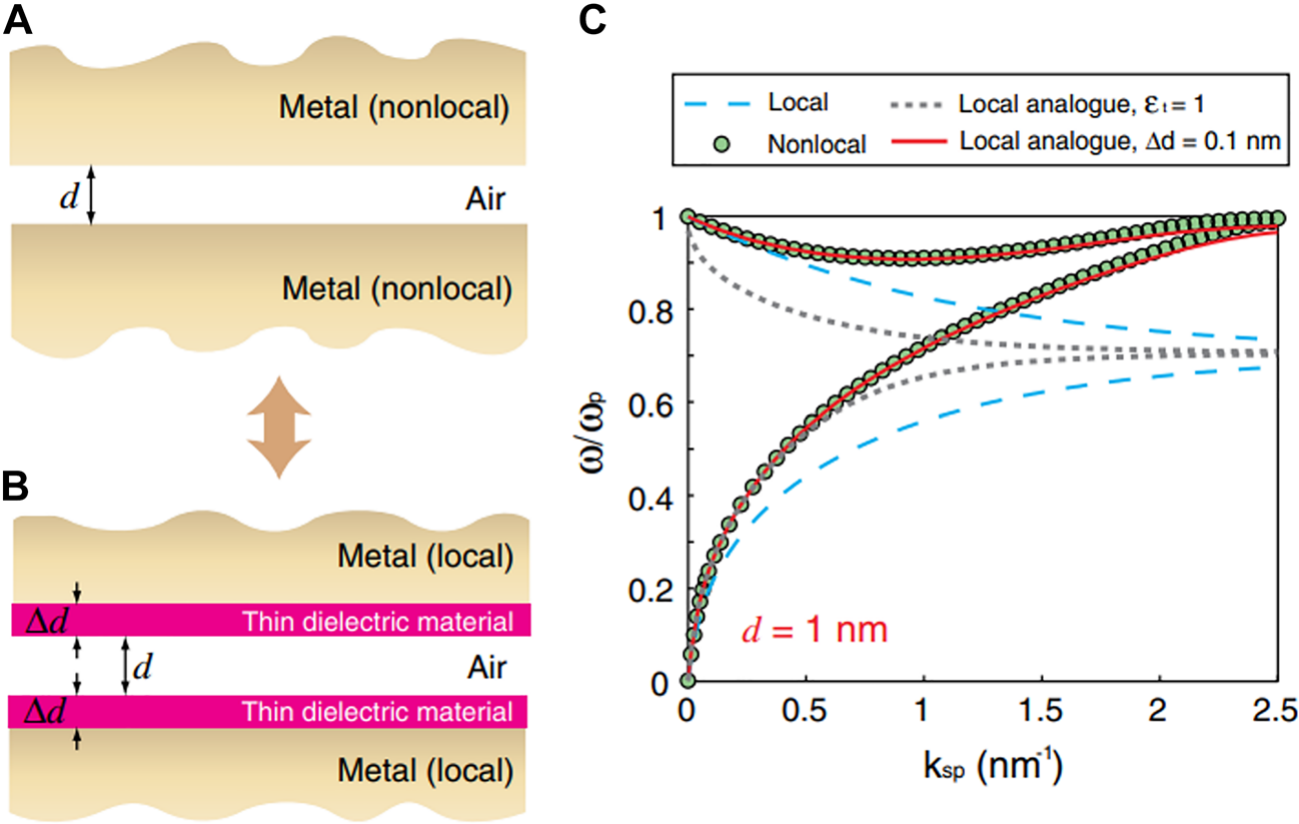
Nonlocal effects in a metallic gap. (A) Metallic gap with nonlocal effects, and (B) modelling of such gaps in local approximation with additional thin dielectric layers. The introduction of dielectric layers may be considered as either simple broadening of the gap $\left(\epsilon_{1}=1\right)$ , or formation of new layers with a fixed thickness (Δd=0.1 nm) . (C) Calculated dispersion relation for the metallic gap in local approximation (cyan dashed), nonlocal picture (filled circle), local analogue with simple gap broadening $\left(\epsilon_{1}=1,\text{gray dotted}\right)$ , and local analogue with new layers (Δd=0.1 nm, red solid) . New layers picture successfully describes the nonlocal effects over the whole frequency range, while simple broadening of the gap can only account for changes in low frequencies. Reproduced with permission from [59].

Regardless of the distance between two metallic layers, nonlocal response of electrons leads to imperfect Thomas–Fermi screening of electric field by metal such that certain amount of electric field normal to the metal–dielectric interface may smear into the metal. For a metal–insulator–metal structure, this effect may widen the ‘effective gap width’, thereby significantly altering the gap plasmon mode formed within the gap. Luo et al. modeled the widening of the effective gap width by introducing additional thin dielectric layers on metallic surfaces and compared gap the plasmon dispersion in a 1 nm-wide gap calculated from full nonlocal calculation and that from a local analogue with the corrections (Figure 4) [59]. From the results obtained near the surface plasmon frequency, the authors conclude that thickness of the dielectric layer Δd=0.1 nm and its dielectric constant is not a constant but depends on dielectric constants of metal, background and longitudinal plasmon normal wave vector. The relation implies that the nonlocal effect should be interpreted as a modification in permittivity of metallic surfaces, and the view of simple widening of the gap is only valid at relatively low frequencies where the metal skin depth is much larger than the surface charge thickness.

**Figure 5:**
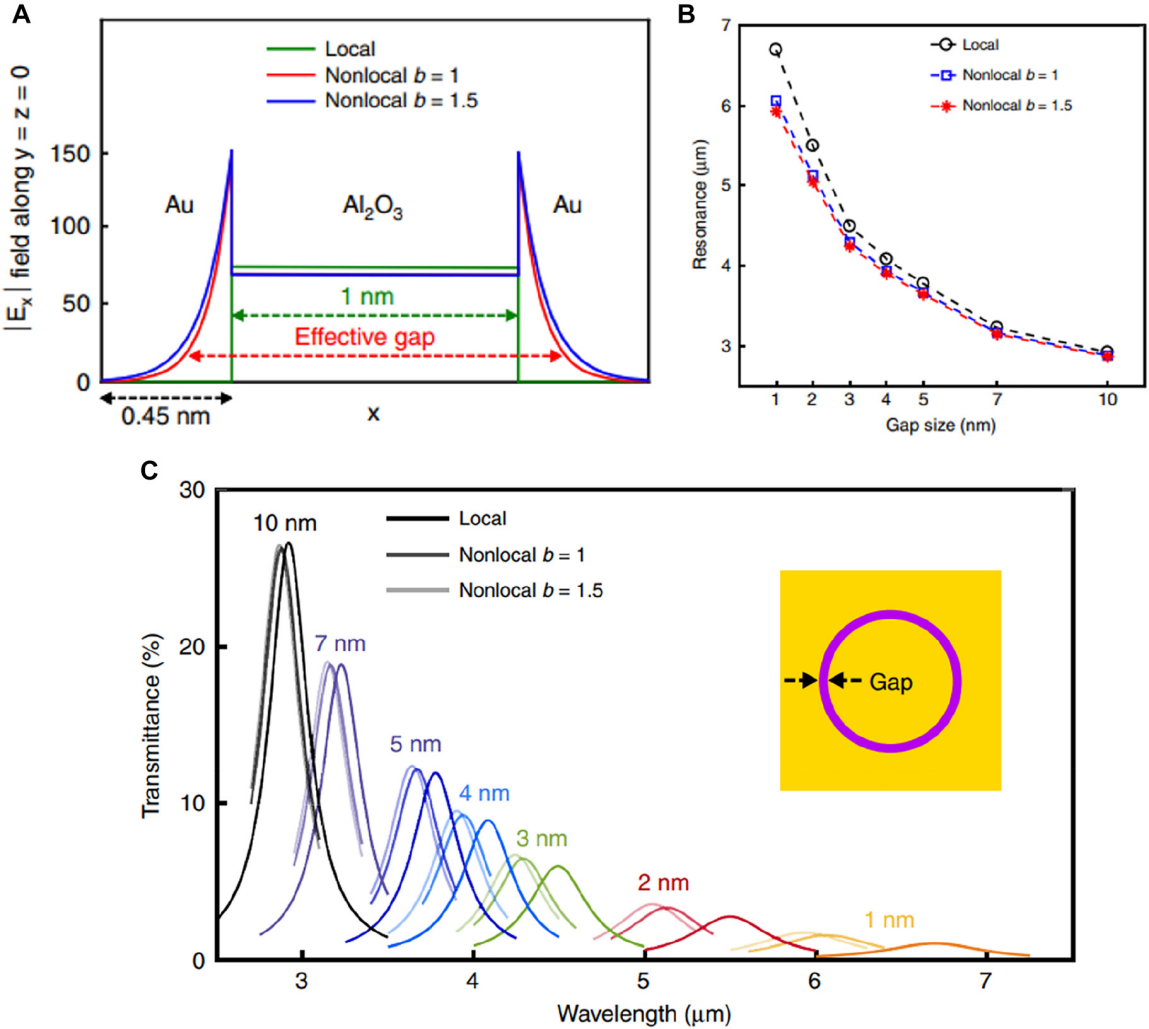
Nonlocal effects in 1–10 nm-wide gaps operating at infrared frequencies. (A) Electric field distribution at vicinity of a 1 nm-wide gap in local and nonlocal pictures. Here, *b* is fitting parameter for the nonlocal effect. (B) Shift in resonance wavelength as a function of gap size, depending on local and nonlocal pictures. (C) Calculated transmittance spectra for different gap widths in local and nonlocal pictures. Note that for gap size smaller than 7 nm transmittance is larger for the nonlocal picture compared to the local counterpart, while for the 10 nm-wide gap the trend is opposite. Reproduced with permission from [60].

**Figure 6:**
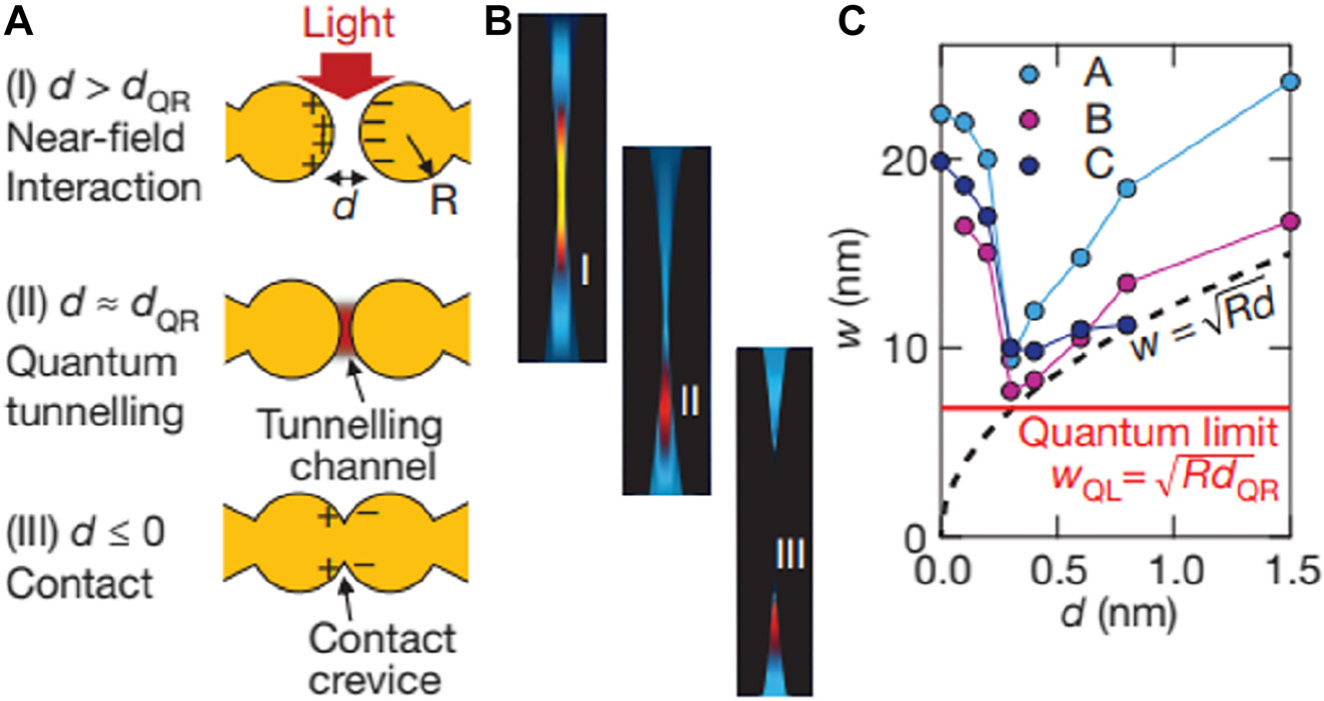
Quantum corrected model for describing sub-nanometer-wide gaps. (A) Three different regimes depending on the gap width. $d_{\text{QR}}$ is the cross-over point where quantum and classical predictions start to diverge. (B) Evolution of a plasmonic mode at the three regimes. (C) Lateral confinement width *w* of three different modes. Quantum tunneling sets a lower bound in mode confinement, as opposed to the classical model where the confinement scales as $w=\sqrt{Rd}$ . Reproduced with permission from [61].

At longer wavelengths where the electric permittivity of a typical metal is orders of magnitude larger than that of dielectrics, the effect of gap plasmon modes is stronger and confinement of the electric field within the gap is nearly perfect. The effect of ‘gap broadening’ is therefore expected to be much more pronounced. In a study performed in mid-infrared by Yoo et al., the authors fabricated and measured transmission spectra of coaxial gaps with gap widths spanning from 1 to 10 nm (Figure 5) [60]. Comparing the experimental results with calculations reveals that the nonlocal effect increases the effective gap width by ∼0.45 nm. The broadening does not depend on the gap width and is only affected by the Fermi velocity of the electrons. Therefore, the nonlocal effect is much more pronounced in smaller (<7 nm) gaps and increases transmission and resonant wavelengths compared to the expected values from local calculations (Figure 6).

For a gap width smaller than ∼1 nm, overlap of electronic wavefunctions from each side of the gap (or meeting of two ‘electronic spill-outs’, in context of the hydrodynamic model) leads to an onset of a charge-transfer plasmon mode which screens accumulated surface charges [62]. The near-field capacitive coupling and correspondingly the field enhancement at the gap therefore decreases significantly, together with a spectral shift in the plasmon modes. In a tip-based geometry with plasmon resonances in visible to near infrared wavelengths, the critical gap width is found to be *d*_QR_ ∼ 0.3 nm, which corresponds to a distance where half of the charge transfer plasmon crosses the gap [61]. It is worth noting that at this regime, the gap is very sensitive to any change occurring within the cavity and exhibit large spectral shifts due to changes occurring in a few-atom scale [63, 64].

**Figure 7:**
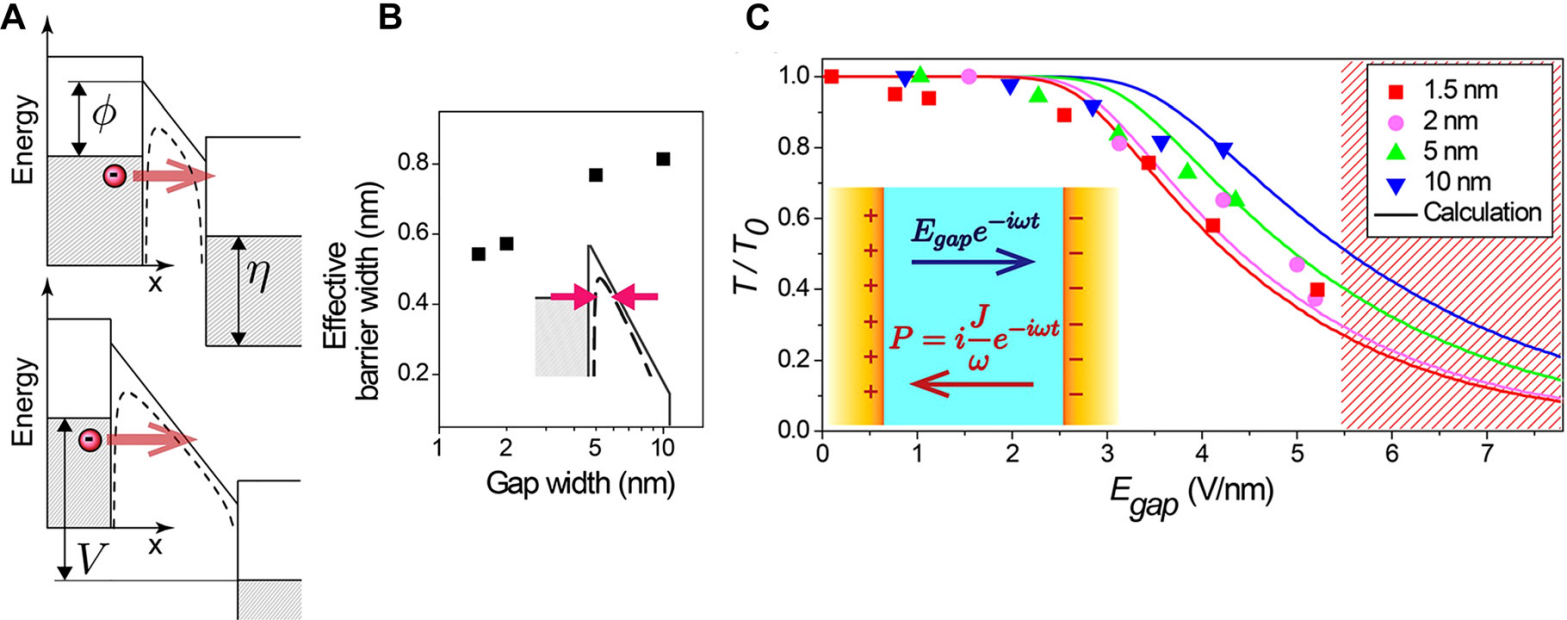
Nonlocal effects in few-nanometer-wide gaps. (A) Tunneling of an electron through a barrier of height ϕ , where dashed lines depict the effective barrier taking the image force into account. With a strong electric field inside the gap $E_{\text{gap}}$ , Fermi level η of the two metals separate by $V=eE_{\text{gap}}w$ which narrows the effective barrier width. (B) Calculated effective barrier widths for different gap widths. (C) Nonlinear terahertz transmittance as a function of $E_{\text{gap}}$ in gaps with different gap sizes due to transient tunneling current $J{\text{e}}^{-i\omega t}$ inducing an additional negative polarization P within the gap. Reproduced with permission from [65].

Lastly, an intense, transient electromagnetic field can also create nonlocal effects on metal–insulator–metal gaps with widths much larger than the critical gap width described above [66] (Figure 7). When then enhanced transient electric field in the gap reaches above ∼2 V/nm, conduction band of the gap-filling dielectric bends towards the Fermi energy of metal and the effective barrier width decreases well beyond the physical thickness of the tunneling barrier. Consequent tunneling current increases nonlinearly as a function of the incident field strength and leads to a nonlinear transmission through the gap. The tunneling effect was observed in 1.5–10 nm-wide gaps operating at terahertz frequencies [65, 67], as well as in a 0.3 nm-wide gap where a single layer of graphene is used as the gap-filling dielectric [26]. It is also worth mentioning that the absence of the gap-filling dielectric may lead to formation of conducting channels between the gaps during tunneling and an eventual breakdown of the gap [68].

### 1.3 Zero-nanometer gap: its definition is frequency-dependent

An effective gap width of ‘zero-nanometer’ implies that two metallic layers comprising the gap are partially touching each other such that electrons can ‘classically’ flow back and forth at the driving frequency, rendering the concept of the gap and capacitance moot. This conduction will, in general, dominate over tunneling or other indirect charge transfer processes in metallic gap structures – for instance, in case of gold, a single quantum of conductance $2/R_{\text{H}}$ dominates over tunneling from an area of 1 m^2^ when the separation between the parallel plates is 2 nm. This will likely change optical properties of the gap dramatically, but in some cases, not so much. For instance, creating a single quantum of conductance through two atomically sharp metallic tips will completely remove the empty space between, and it would be enough to make the gap width ‘zero-nanometer’. For millimeter-long slot antennas operating at terahertz frequencies, a single point of conduction will reduce charge accumulation only partially, such that there will be only minute decrease in the field enhancement and transmission through the gap. In this section, we will discuss the case of extended gap structure and try to define ‘zero-nanometer’ regime in such systems.

**Figure 8:**
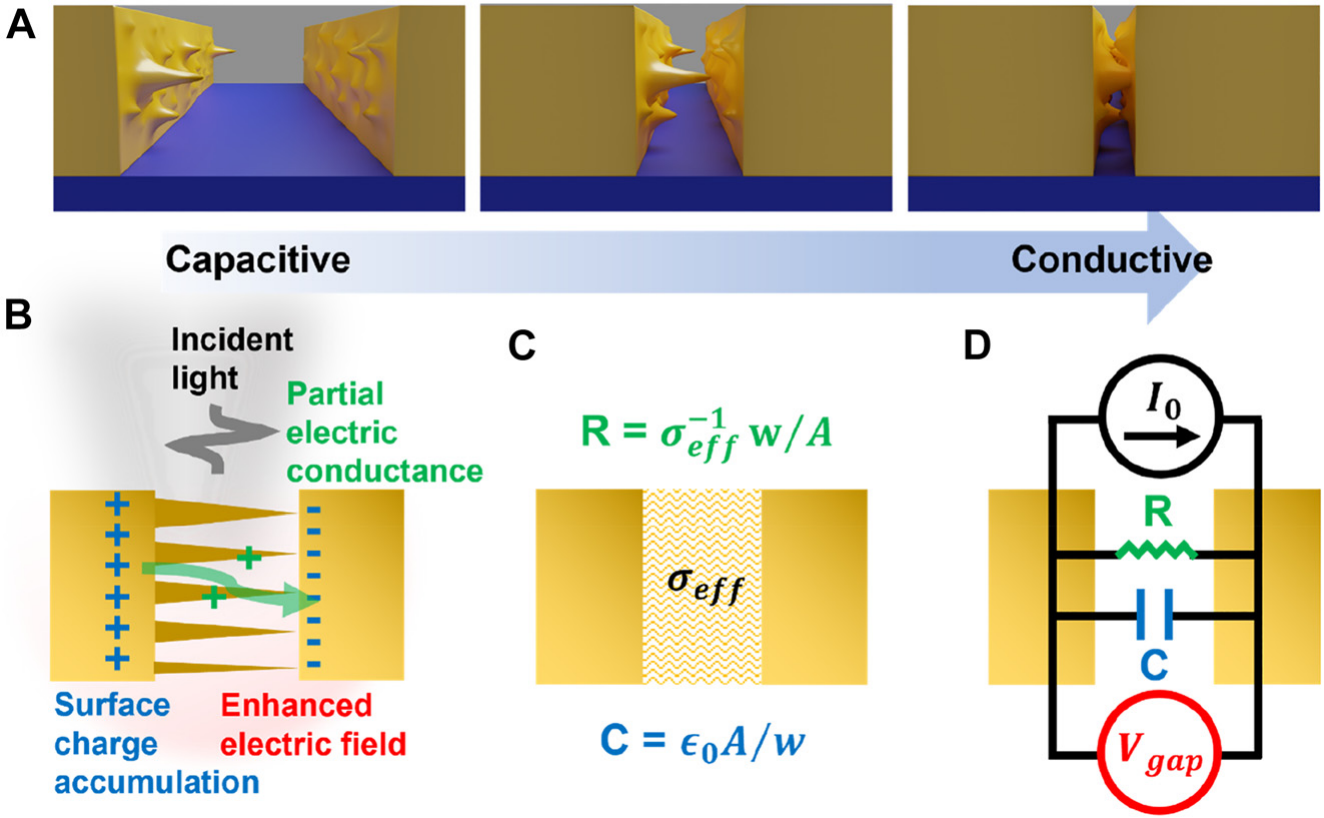
Zero-nanometer limit for an extended gap. (A) A schematic view of an extended metallic nanogap reaching a subnanometer width and below. The gap does not close uniformly, and there always exist salient *pyramids* that make point contacts first. The gap therefore gradually transitions from capacitive to conductive screening regime. (B) Description of an extended gap in a partially conducting regime. Incident light creates surface current which in turn accumulate charges at the surface and induce the electric field enhancement. The capacitive charge accumulation is partially screened by conduction of electric current through the pyramids. (C) Effective medium description of the gap depicted in (B). Permittivity of the gap $\epsilon_{\text{gap}}$ is related to $\sigma_{\text{eff}}$ via Eq. (14) in the main text. (D) *R*–*C* circuit representation of the gap, where each component is drawn in the same color as the corresponding phenomenon in (B). Response of the circuit can be described as $V_{\text{gap}}\left(f\right)=I_{0}\left(f\right)/\left(R^{-1}-i2\pi fC\right)$ , which allow us to express the effective optical permittivity of the gap as $\varepsilon_{\text{gap}}=1+i/2\pi fRC$ .

When attempting to reach zero-nanometer in extended gap structures, we can imagine that the gap will not close uniformly and there always exist salient conducting pyramids that make contact first. As the gap width becomes smaller, number of the conducting channels will increase, and more electrons will conduct instead of being accumulated on the surface. Such formation of atomically thin conducting channels in extended gap structures manifests itself as jumps in conductance between the two metallic layers, as shown in Figure 8. At a certain value of total conductivity, the structure will show negligible charge accumulation and optically behave the same as a bare film, which may be called the ‘zero-nanometer’ limit for extended gap structures.

In the following discussion, we will try to determine the limit by describing the partially conducting gap as an equivalent RC circuit. For the sake of simplicity, we will consider an infinite slit such that the gap is purely capacitive. When an electromagnetic radiation is incident onto the metallic structure, surface current is created per Maxwell boundary condition $\Delta H_{∥}=K$ . This will induce surface charges to accumulate in a capacitive manner and incorporate an electric field enhancement at the gap. When there are several conducting channels, a portion of the accumulated surface charges will flow through the gap and partially screen the capacitive charge accumulation. Therefore, the interaction of the gap with an incident electromagnetic field can be described as an *R*–*C* circuit connected to a constant current source, where voltage difference across the gap (a capacitor with capacitance *C*) stands for the enhanced electric field in the gap and the resistance *R* stands for the conducting channels formed by the multiple pyramids. Under such *R*–*C* modeling, we may express voltage difference across the gap $V_{\text{gap}}\left(t\right)$ as

(10) $$V_{\text{gap}}\left(t\right)=\frac{1}{C}\int_{-\infty }^{t}{\text{e}}^{-\frac{t-\tau }{RC}}I_{0}\left(\tau \right)\text{d}\tau ,$$

which convolutes into the following frequency domain equivalence equation,

(11) $$V_{\text{gap}}\left(f\right)=\frac{I_{0}\left(f\right)}{\left(\frac{1}{R}-i\cdot 2\pi fC\right)}=\frac{RI_{0}\left(f\right)}{1-i\cdot f/f_{C}},$$

where $f_{C}=1/2\pi RC$ . This simple relation implies that the voltage across the gap in the presence of conducting channels differ dramatically depending on the operating frequency. For large frequencies $f\gg f_{C}$ , the gap is mostly capacitive, and the effect of resistance is not as important. In the regime $f\ll f_{C}$ , the gap is now dominated by the 1/R term, such that formation of only a few conducting channels dramatically affects the field enhancement at the gap. Therefore, an extended metallic gap operating at far infrared or longer wavelengths is expected to reach the regime of ‘zero-nanometer’ with much smaller number of conducting channels compared to the gaps operating at visible or near infrared wavelengths.

Now, we try to make connection between these *R*–*C* parameters and an effective optical permittivity of the gap. We consider multiple conducting channels formed across a gap with area *A* and width *w*. These channels are connected parallelly such that the total resistance across the gap can be expressed as

(12) $$\frac{1}{R}=\overset{N}{\underset{i=1}{\sum }}\frac{2}{R_{\text{H}}}m_{i}\left(w\right)=\frac{2N}{R_{\text{H}}}m\left(w\right)=\frac{\sigma_{\text{eff}}A}{w},$$

where *N* is the number of point contacts between the two surfaces, $R_{\text{H}}=h/e^{2}$ is the inverse of quantum Hall conductance, $m_{i}\left(w\right)$ is a weight function for the *i*th channel, and $\sigma_{\text{eff}}$ is the effective conductivity of the channels. Note that area of each contact may differ depending on shapes of the pyramids and the gap width, and that such effects are accounted for by the weight function $m_{i}\left(w\right)$ . Since $C=\varepsilon_{0}A/w$ for an empty gap, we may write an expression for the imaginary part of the effective permittivity inside the gap $\varepsilon_{\text{gap}}$ as

(13) $$\text{Im}\left(\frac{\varepsilon_{\text{gap}}}{\varepsilon_{0}}\right)=\frac{1}{\omega RC}=\frac{\sigma_{\text{eff}}}{\varepsilon_{0}\omega }.$$

When there is finite damping in the system, we may write

(14) $$\frac{\varepsilon_{\text{gap}}}{\varepsilon_{0}}=1+i\frac{\sigma_{\text{eff}}\gamma }{\varepsilon_{0}\omega \left(\gamma -i\omega \right)}.$$

The above approach was successfully used to describe terahertz transmission through nanoslots with transient tunneling current through the gap [69]. By assuming that conducting channels are randomly dispersed throughout the whole gap and that dimensions of each channel are much smaller than the wavelength, we may follow the approach above and consider the partial connections throughout the gap as a homogeneous medium with an effective conductivity $\sigma_{\text{eff}}$ .

**Figure 9:**
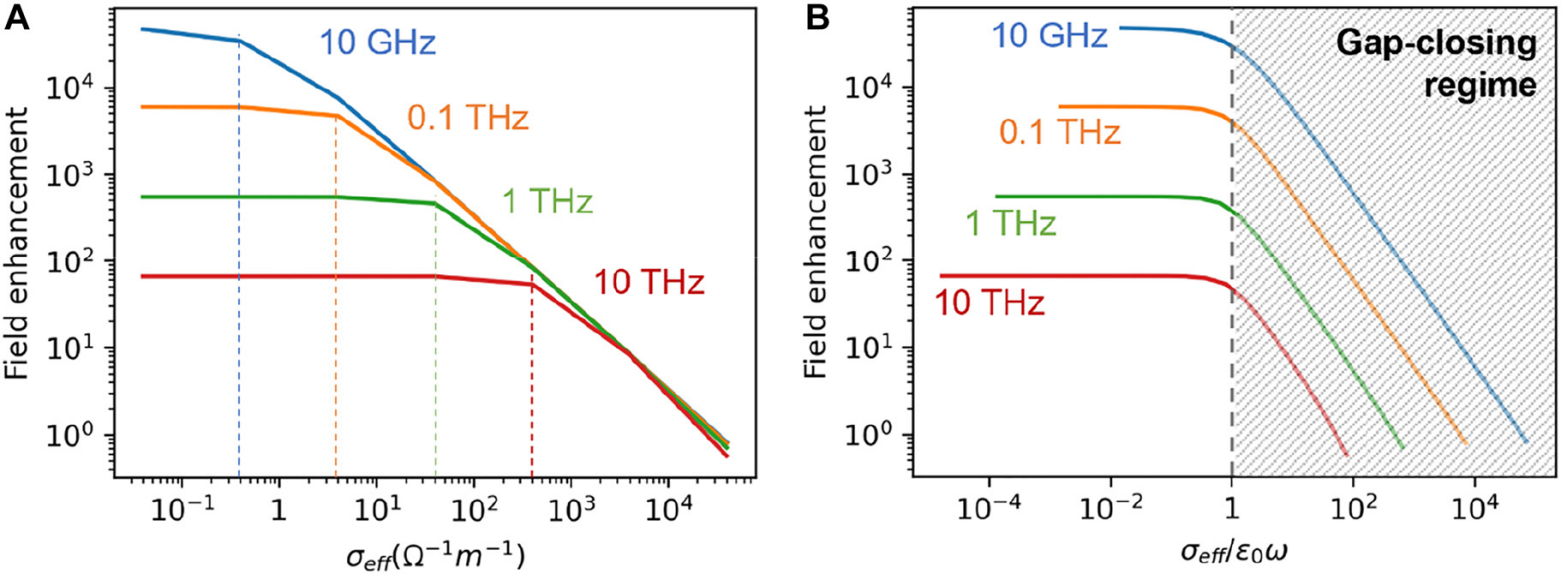
(A) Field enhancement factors of an infinite slit array with 1 nm gap width and 50 μm period, filled with effective conductivity $\sigma_{\text{eff}}$ . Deflection points appear at different values of $\sigma_{\text{eff}}$ for different frequencies. (B) Field enhancement factor as a function of $\sigma_{\text{eff}}/\varepsilon_{0}\omega$ for different frequencies, showing universal transition behavior at $\sigma_{\text{eff}}/\varepsilon_{0}\omega ≃1$ .

Figure 9A shows calculated electric field enhancement factors of infinite slit arrays with gap width w=1 nm and period p=50 μm fabricated on a free-standing gold film of thickness h=200 nm , with a gap-filling medium with different values of $\sigma_{\text{eff}}$ . For lowest values of $\sigma_{\text{eff}}$ , the slits efficiently accumulate charges in capacitive manner, and the field enhancement scales inversely with the frequency. For larger values of $\sigma_{\text{eff}}$ field enhancement decreases rapidly as the slits become more like a homogeneous thin film of a Drude metal, and the field enhancement becomes constant over a broad range of frequencies. Most importantly, the start of the zero-nanometer limit, indicated by onset of the drastic decrease of the field enhancement, is further and further delayed as we go to the higher and higher frequencies. Therefore, for a fixed number of conducting channels it is generally easier to achieve ‘zero-nanometer limit’ in lower frequencies.

When we plot the field enhancement as a function of $\sigma_{\text{eff}}/\epsilon_{0}\omega$ instead, we find a universal behavior where transition to the ‘zero-nanometer gap’ starts almost exactly when $\sigma_{\text{eff}}/\epsilon_{0}\omega ≃1$ . This may be interpreted as the point where imaginary part of the effective permittivity of the gap starts to overwhelm the real counterpart. For an operating frequency of 1 THz, the onset starts at σeff≃56 Ω−1 m−1 , which is approximately six orders of magnitude smaller than conductivity of bulk gold. This is $7.22\times 10^{7}G_{0}$ per 1 cm^2^ area within the gap, and this converts to a real coverage ratio of conducting channels of $1.2\times 10^{-9}$ , assuming a single atomic contact per $G_{0}$ . It should be noted that the above deducted value only provides a lower bound; nevertheless, it is obvious that very small number of electric contacts through the gap can completely shut off long wavelength radiations, as was partially demonstrated in slot antennas with an embedded metallic nanorod [70, 71]. In general, this is a great disadvantage as it indicates that the gap is prone to external stimuli. However, for certain types of gap geometry this becomes a big advantage and calls for many possible applications in millimeter and microwave frequencies, which will be discussed in Chapter 3 in more detail.

## 2 Experimental realization of sub-to-zero nanometer gaps

Development of nanophotonics has always moved along with advances in nanofabrication technologies. Resolution of the current state-of-the-art lithography is 3–5 nm, mostly limited by secondary scattering of energetic particles as in focused ion beam milling (Ga or He ion) and electron beam lithography (electrons). These methods are also not well suited for fabricating nanostructures in wafer-scale. To overcome the limit and reach the regime of ∼1 nm and below, various strategy have been developed. In this Chapter we will briefly overview well-known methods for fabricating sub-10 nm gaps and introduce various means to push the limit to sub-to-zero nanometers. Classical yet still widely used methods include tip-based, feedback-controlled approach and particle-on-a-mirror geometry with a very thin dielectric spacer, which lead to a vertically aligned MIM structure. In-plane MIM structures can also be fabricated with a spacer-based method and can have gap widths as small as 0.3 nm. To reach the limit of zero-nanometer, cracks and breakjunctions are a natural choice. Recent advances in fabrication methodologies have also enabled formation of lithographically prepatterned, extended breakjunctions referred to as ‘zerogaps.’

### 2.1 Established methods for sub-10 nm to few-nanometer gaps

The most well-established methods for fabricating sub-10 nm-wide gaps are extreme ultraviolet (EUV) photolithography, electron beam lithography (EBL), and focused ion beam (FIB) milling [72]. These share a similarity in that they all use energetic particles to change the material property (of a photoresist) or directly etch the target material. In EUV photolithography, an intense EUV light with 13.5 nm wavelength is used to project patterns in photomask onto a photoresist coated on a wafer. The method can create patterns with feature size smaller than 10 nm over a whole wafer and is being used in the state-of-the-art semiconductor manufacturing. Major drawback of EUV photolithography is that the system is very costly (∼$100 M) due to extremely strict requirements on roughness and size of mirrors, intensity of EUV light source, etc. More affordable alternatives are EBL and FIB which use electrons and gallium ions, respectively, to either modify chemical state of the resist or to directly etch the material of interest. While their resolution varies from 100 to few tens of nanometers, accounting for proximity effects in EBL and using helium ions for FIB can greatly reduce the minimum feature size [73], [74], [75]. These are the most widely used methods for fabricating nanophotonic structures such as bowtie antenna, nanohole array, etc. and gap widths of 1 nm or less were achieved [76], [77], [78]. However, due to a limited field of view and long operation time, these methods are not well suited for wafer-scale fabrication or creating gaps for long wavelength applications. In the following section, we will mainly discuss schemes that are well suited for wafer-scale fabrication; we briefly note that there are many other fabrication methods such as intragap nanoparticle synthesis [7, 79], [80], [81], [82], intergap nanoassembly [83], [84], [85], [86] and nanoparticle-on-a-mirror [87], [88], [89], [90], [91], [92], [93], [94] if we do not limit ourselves to the large area gaps.

**Figure 10:**
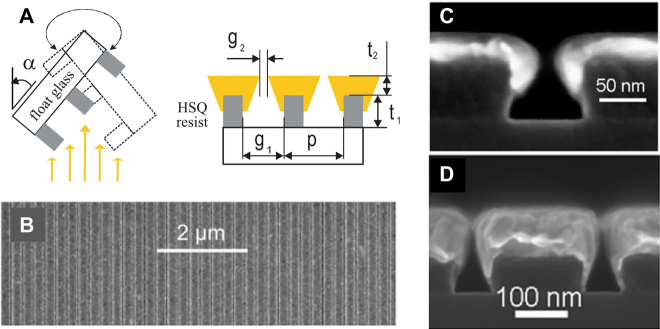
Nanogap fabrication with shadow evaporation technique. (A) Schematic image of the method and parameters that determine the final gap width. (B) Top view SEM image of the fabricated nanogap array. (C) and (D) Cross-sectional SEM images of fabricated nanogap arrays with different gap widths. Reproduced with permission from [95].

To fabricate sub-10 nm gap structures over a large area, various methods have been developed. One of the most classical method is shadow evaporation, also called glancing angle evaporation [96] (Figure 10). In this approach, a relatively coarse grating pattern is first realized with photoresist on a substrate by other lithographical means. Then the sample is mounted on a metal evaporator at a glancing angle and rotates at a constant rate such that evaporated metal coats the resist uniformly. The minimum gap width is determined as initial separation between the resist patterns minus two times the thickness of deposited metal. Siegfried et al. fabricated 5 nm-wide slit arrays [95], and Theiss et al. fabricated nanoparticle dimers with 1 nm separation using this method [97]. A variation of this method uses closely packed monolayer of nanospheres as the mask and is also proven to be effective in creating sub-10 nm gaps over a large area. With this method, generally referred to as nanosphere lithography, Ji et al. fabricated sub-10 nm gaps and controlled the width by partially dry-etching the spheres to reduce their sizes and therefore empty space between them [98]. Controlling thickness of deposited metal leads to transition from arrays of metallic patches to nanohole arrays which may find different applications [99]. It is worth mentioning that while this method is well suited for mass fabrication of nanogaps, it is also capable of creating point-gaps such as nanoparticle dimers [100]. Overall size of the nanostructures fabricated with this method is several hundred nanometers and they find uses in surface enhanced Raman spectroscopy (SERS) or super-resolution imaging, etc. [101, 102]. Despite such versatility, shadow evaporation is not well suited for reducing the gap size to below a few nanometers due to diffusion of metal atoms accidentally creating contacts through the gap.

**Figure 11:**
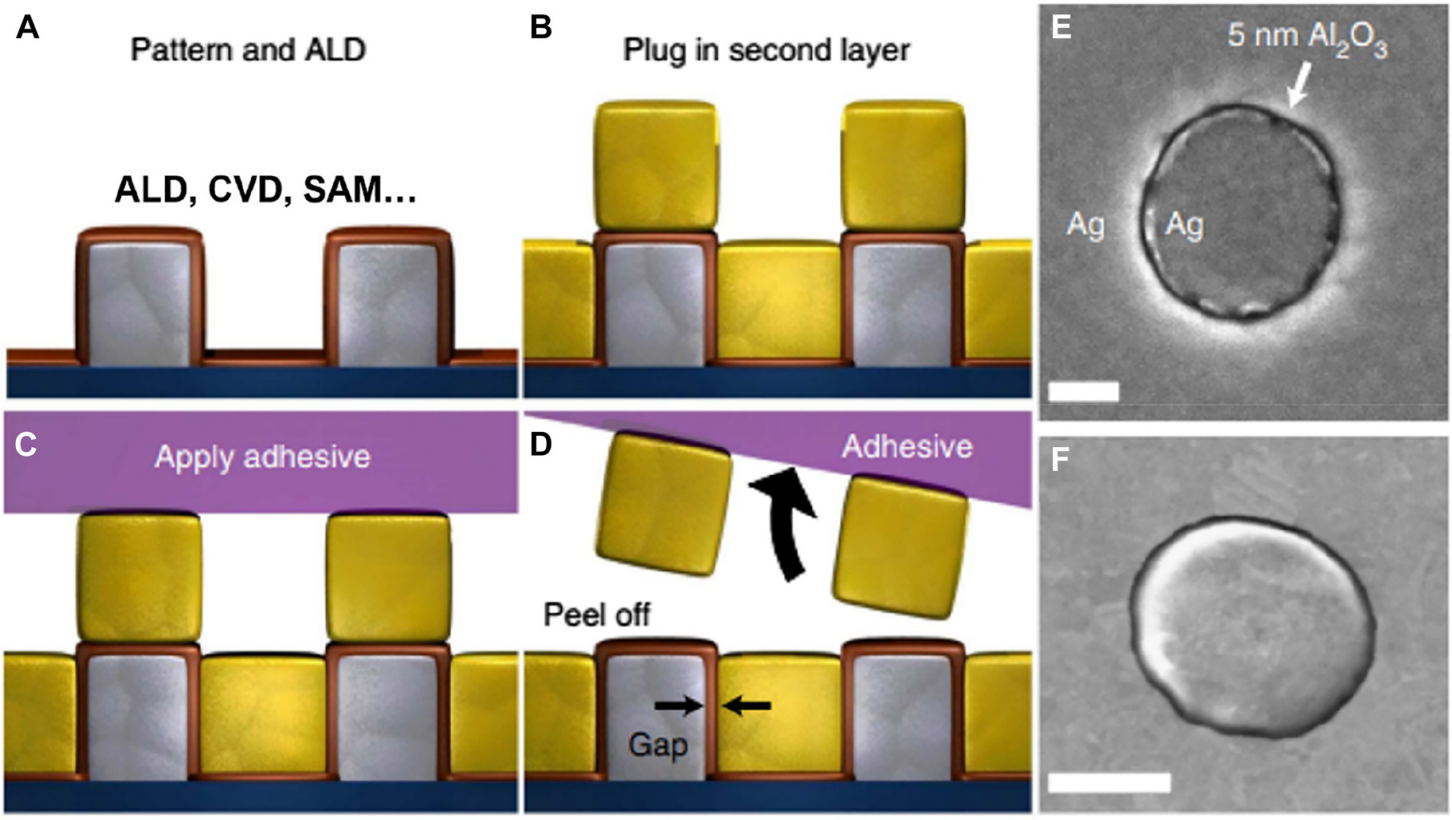
Nanogap fabrication with atomic layer lithography. (A) First metallic pattern is conformally coated with a dielectric layer with either atomic layer deposition (ALD), chemical vapor deposition (CVD), or self-assembled monolayer (SAM). (B) Second layer of metal is deposited on top of the whole sample, filling the empty space, and defining in-plane-stacked metal–insulator–metal structure. (C) and (D) Applying adhesive and peeling off the overhanging metal layer planarizes the sample and reveals the gap. (E) and (F) Top view SEM images of fabricated gap structures, whose gap width is determined by the thickness of deposited dielectric layer. Scale bar: 300 nm (E) and (F). Reproduced with permission from [25].

Another way to fabricate sub-10 nm metallic gaps in a more reliable manner is to use a dielectric spacer between two metallic layers to make a metal–insulator–metal (MIM) structure. The dielectric layer prevents accidental connection between the two metallic layers such that the gaps can be fabricated over a large area with a high throughput. Also, as deposition of dielectric layers can be controlled with a monolayer accuracy depending on the material and deposition method, the gap width can be controlled precisely down to ∼1 nm accuracy. In 2006, Miyazaki et al. used sequential deposition of gold and silicon oxide films to realize an out-of-plane-stacked, 3 nm-wide metal–insulator–metal gap structure, but the gap cannot naturally couple to electromagnetic radiation incident normally onto the substrate [103]. Using ‘nanoskiving’ to slice the gap may enable re-orientation of such gaps and stacking of the gap structures in 3-dimensional architecture, but thickness of the metal cannot be controlled precisely [104, 105].

Chen and Park et al. showed that it is possible to fabricate *in-plane-stacked* metal–insulator–metal gap structure if the gap-filling dielectric is deposited with atomic layer deposition (ALD), as it is capable of depositing dielectric films in isotropic manner with thickness precision of ∼1 nm [25] (Figure 11). Fabrication procedure for this method, usually referred to as atomic layer lithography, is as follows. First, a metallic pattern is created with standard photolithography or e-beam lithography. Next, aluminum oxide is deposited with ALD and covers the whole wafer including the sidewalls of the first metallic pattern. Then a second layer of metal is deposited on top of the whole structure, defining vertically aligned metal–insulator–metal gap on the sidewall. Overhanging excess metal is then removed via exfoliation to planarize the whole structure and to expose the gap. Detailed shape of the gap opening may be controlled by choice of different resists, metal thicknesses, and substrates [106]. Note that perimeter of the first metallic pattern determines the length of the gap, such that there is practically no limit in achievable length and therefore operating wavelength. Metallic gaps with widths spanning from a few tens to one nanometer have been realized with this method and have been utilized in various range of wavelengths spanning from visible, infrared, terahertz [39, 107], [108], [109], [110], [111] and even microwaves [112]. The ALD may be replaced with other coating processes such as self-assembled monolayer (SAM) [113] or other types of chemical vapor deposition (CVD), and an ultimate gap width of a single atom thickness (0.3 nm) has been realized with this method by choosing CVD-grown graphene as the dielectric [26]. There are also variants to this method depending on the method of planarization, for example, chemical etching of a sacrificial layer [114], ion milling at a glancing angle [11, 115], and templated stripping [116], etc.

**Figure 12:**
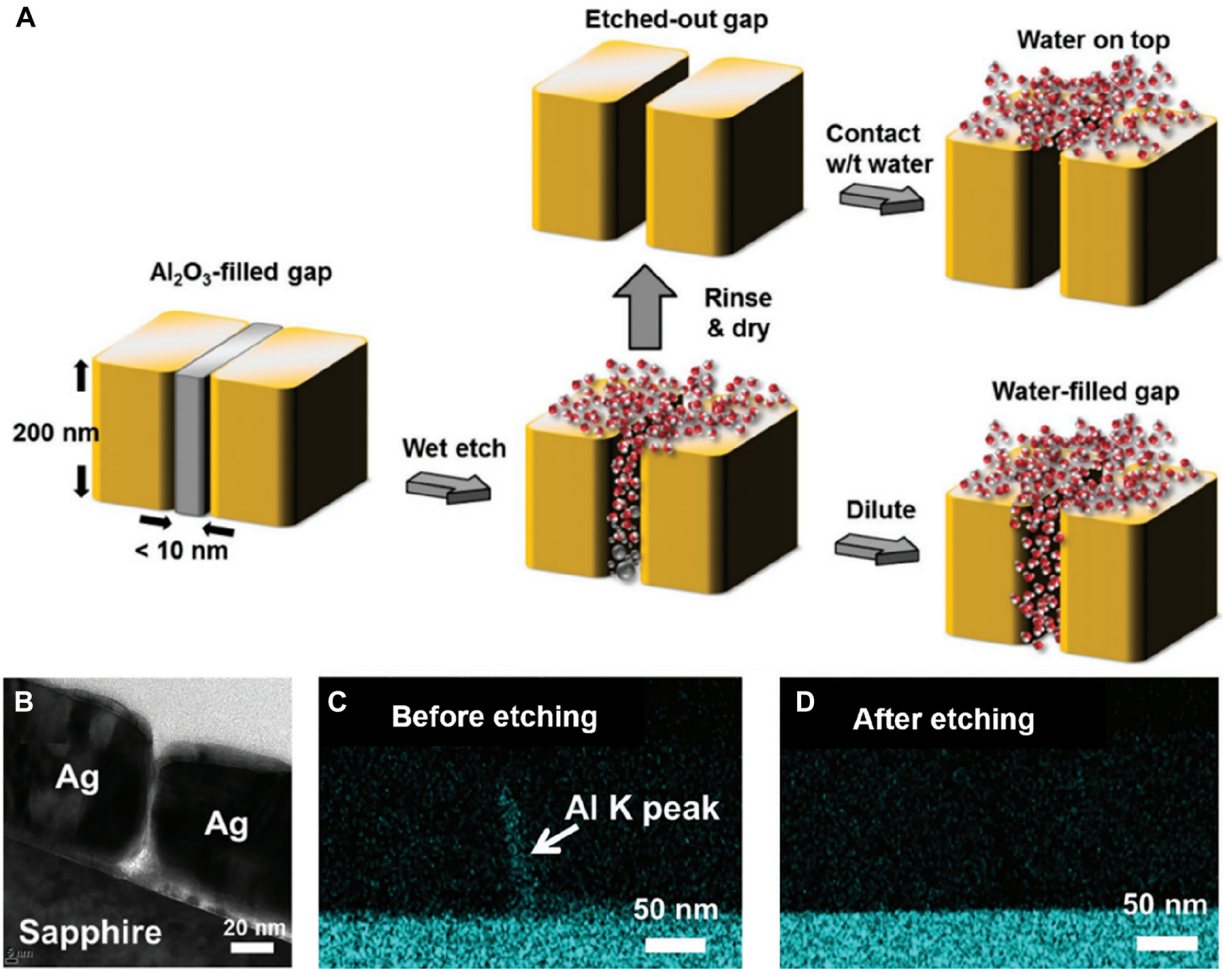
(A) Wet-etching the gap-filling dielectric in metal–insulator–metal (MIM) gap. While water and etchant molecules cannot enter an empty metallic gap due to surface tension, they can enter a gap filled with dielectric as the reaction between the etchant and the dielectric is thermodynamically favored. (B) Cross-sectional transmission electron microscope (TEM) image of a few-nanometer gap retaining its shape after the wet etching process. (C) and (D) Energy-dispersive X-ray spectroscopy mapping of an MIM gap before and after etching, showing successful removal of gap-filling aluminum oxide. Reproduced with permission from [117].

The biggest limitation of the spacer-based metallic gaps is that the ‘hot spot’ – where the electromagnetic field enhancement is the strongest – is already filled with the spacing dielectric such that it cannot be efficiently coupled to other systems of interest. One way of circumventing the issue is to use the target material itself as the spacer – Tripathi et al. utilized assembled monolayer of quantum dots as the spacer to fabricate extended metallic nanogap structures that can operate at both terahertz and visible frequencies [118, 119]. Still, this method can be applied to limited sets of materials, and the gap should ideally be empty for integration with various material systems. Jeong et al. showed that an empty gap can be realized by carefully wet-etching the dielectric layer in a 10 nm-wide metal–insulator–metal gap [117] (Figure 12). As reaction of the dielectric and wet etchant is thermodynamically favored, the etchant and water can enter the metallic gaps despite hydrophobicity of noble metals. After the dielectric is fully etched and the gap is filled with water molecules, the hot spot can be filled with other materials including alcohols and dye molecules via diffusion and dilution. Full etching of the gap-filling dielectric can be confirmed by energy dispersive X-ray spectroscopy (EDS) mapping of the cross-sectional transmission electron microscope (TEM) image, and from spectral shifts in terahertz transmission upon etching and filling the gap. By using critical point dryer (CPD) for removing the gap-filling water after the wet-etching, catastrophic collapse of gap due to surface tension can be prevented and empty gaps with widths as small as 1.5 nm could be achieved [120]. It is also worth mentioning that the gap fabricated this way can be further narrowed by factor of ∼2 utilizing thermal expansion of metals when the gap width is orders of magnitude smaller than periodicity or overall size of the metallic layer [108].

### 2.2Out-of-plane-oriented point gaps: tip-based approach

Tip-based approach can create metallic gap by feedback-controlling distance between tip probe and substrate surface and is by far the most accurate means to achieve sub- to zero-nanometer gaps. Two geometrical features of the tip-based gap induce interesting nano-optical effects that are favorable to localization of electromagnetic wave within extremely small volume between the tip apex and the surface. Incident electromagnetic wave aligned along the probe axis drive the free charge of metallic probe into the sharp probe apex (lightning rod effect) enhancing the electric field intensity localized by surface plasmon polariton. Also, capacitance gap between the tip and the substrate surface plays a vital role in electromagnetic field localization by funneling the electric field into the gap. Tip-based gap structures are frequently implemented in tip enhanced Raman spectroscopy (TERS) facilitating the enhanced gap plasmon mode enabling diffraction-limit free spectroscopy [121], [122], [123], [124], [125].

Electric field polarization is important issue in tip-based gap structure-based spectroscopy where localization of maximum electric field intensity in the gap is highly sought [126, 127]. To fulfill the electric field polarization condition parallel to tip axis, illumination of excitation laser in the oblique angle with TM polarization [128], or focusing of radially polarized light incident along the tip axis [129, 130] have been used in with tip-based nanospectroscopy. On the other hands, electric fields polarized along nonparallel to tip axis can contribute to obtain maximum spectroscopic signal depending on molecular orientation of analyte confined on the substrate [131], [132], [133] or geometry of nonplanar metallic substrate which would have complex electric field vector profiles [134]. Various tip geometries have been demonstrated to provide the preferred electric field polarization at the tip apex, vertical or parallel to tip axis, including brunt metal coated probe [135, 136], split-ring resonator mimic probe [137], and gold particle attached probes [134, 138].

**Figure 13:**
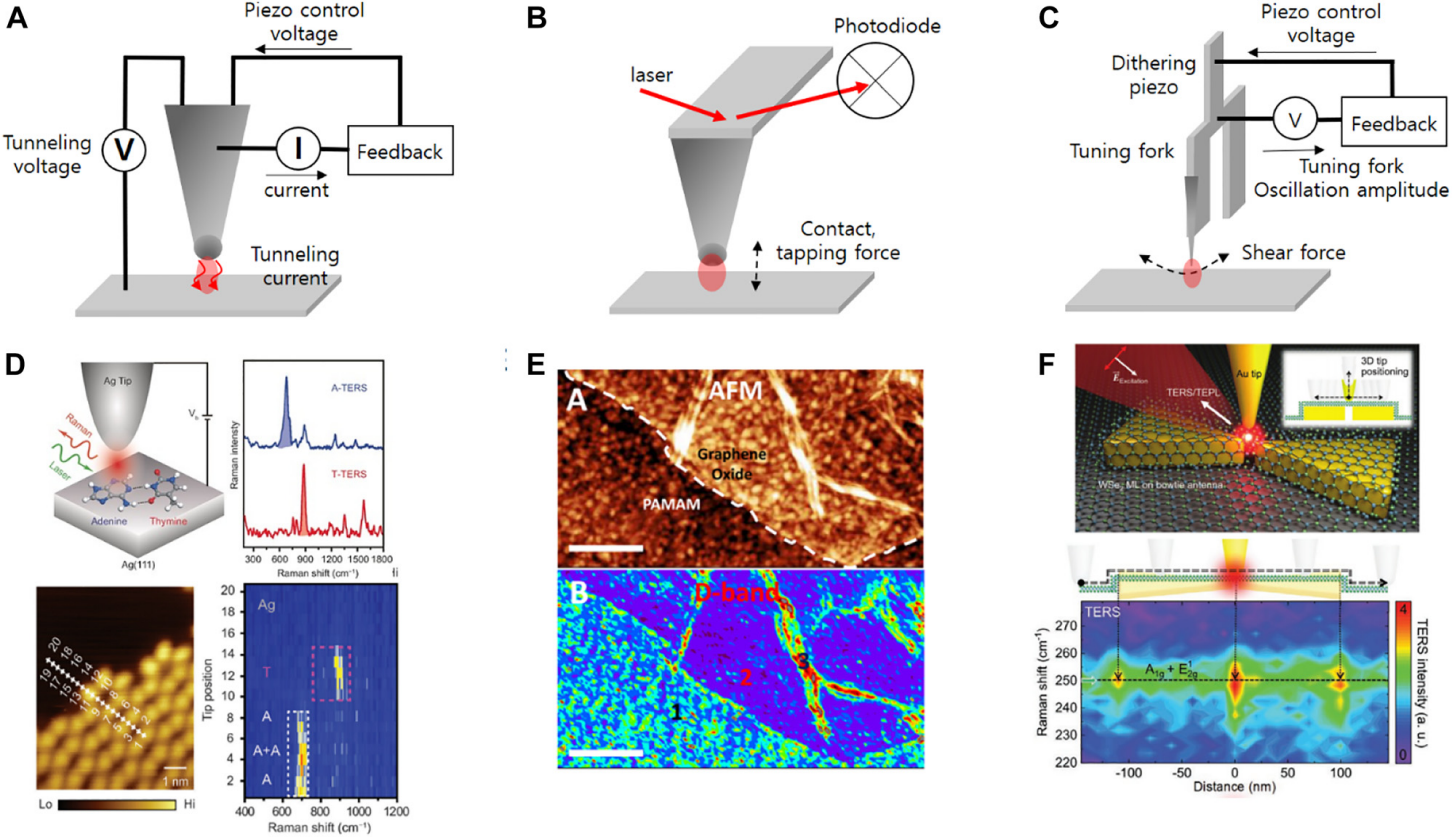
Tip based zerogap (TZ) geometries depending on feedback mechanism for tip-sample surface distance control. (A) TZ of tunneling current feedback controlled STM probe. (B) TZ of AFM controlled by cantilever deflection magnitude feedback mechanism. The cantilever deflection is monitored by laser intensity reflected from cantilever top surface. (C) TZ of shear-force feedback controlled metallic probe. (D) Single strand DNA sequencing demonstrated by STM based TERS [139]. (E) Hyperspectral Raman image of Graphene 2D material obtained by AFM based TERS [140]. (F) Local excitation of exciton of TMD 2D material coupled to bowtie nanostructure and shear-force controlled metallic tip probe [31].

The gap distances are controlled by feedback mechanisms typical of atomic force microscope (AFM) and scanning tunneling microscope (STM). STM facilitates tunneling current between conductive probe and the substrate which are strongly affected by the gap distance between them enabling gap width control in tens of picometers range (Figure 13A). Under the low temperature and vacuum operation conditions, STM gap enables the single molecular level acquisition of electron density mapping and Raman hyperspectral images [141, 142]. Spatial resolution of STM reaches atom and molecular levels as demonstrated in sequencing of single DNA helix (Figure 13D) [139].

AFM controls the tip-sample surface distance by measuring the light deflection from the cantilever which supports the probe. The magnitude of the deflection is monitored by measuring the laser intensity reflected from the tip cantilever. The magnitude of cantilever deflection can be converted to the atomic force between tip apex and the sample surface, which can be facilitated for feedback control of gap distance of AFM in a few-nanometer range. AFM-based gap can be used under the ambient condition because the feedback signal for gap width control (laser intensity) does not require vacuum. In addition, AFM can be applied to samples with height differences of a few micrometers, which is much larger than those in STM. Above mentioned advantages of AFM-based gaps enable TERS and spatially resolved PL measurement of 2D materials including graphene and transition metal dichalcogenides monolayers [140, 143].

Feedback control using shear force between laterally oscillating probe and sample surface can be implemented by attaching sharp metallic probe onto dithering piezo components such as quartz tuning fork. Shear-force feedback-controlled gap can be operated in room temperature and atmospheric pressure condition, and gap distance of 2–3 nm can be maintained. Equipped with chemically etched metallic sharp tip probe, which is also used in STM, shear-force feedback-controlled gap enabled highly efficient light–matter interaction. Recently, local excitation of excitons in 2D material at the adjacent area between sharp Au metallic probe and bowtie shape nanostructure have been demonstrated by using shear-force feedback-controlled gap [31]. Such great controllability in gap width, and position of sub- to zero-nanometer gap makes a tip-based gap an ideal geometry for spatially resolved spectroscopy. It should also be noted that the tip-based gap can be applied to infrared and terahertz frequencies as well [144], [145], [146], and pump–probe type experiments are also being performed for nanoscale mapping of ultrafast dynamics [147, 148].

### 2.3 In-plane-oriented point-gaps: breakjunctions

As the tip-based approach requires extreme care, it is not suitable for mass manufacturing and device applications. The gap is also aligned out-of-plane, which hinders natural coupling of the gap with the incident light. To achieve gap widths smaller than the lithographic limit, it is natural to create the gap from ‘zero-nanometers’, rather than trying to decrease it from a few nanometers. Such approach was first made in the field of molecular electronics, where a break junction is utilized as a pair of electrodes with nanometer separation [149], [150], [151], [152], [153], [154]. In this method, a part of the metallic structure is selectively thinned down by means of shadow evaporation technique or focused ion beam milling, etc., and then disconnected by applying additional external stimuli, leading to a point metallic gap with an effective area as small as ∼1 nm^2^. Formation of the junction can be observed in real time by monitoring the jump in resistance across the wire.

**Figure 14:**
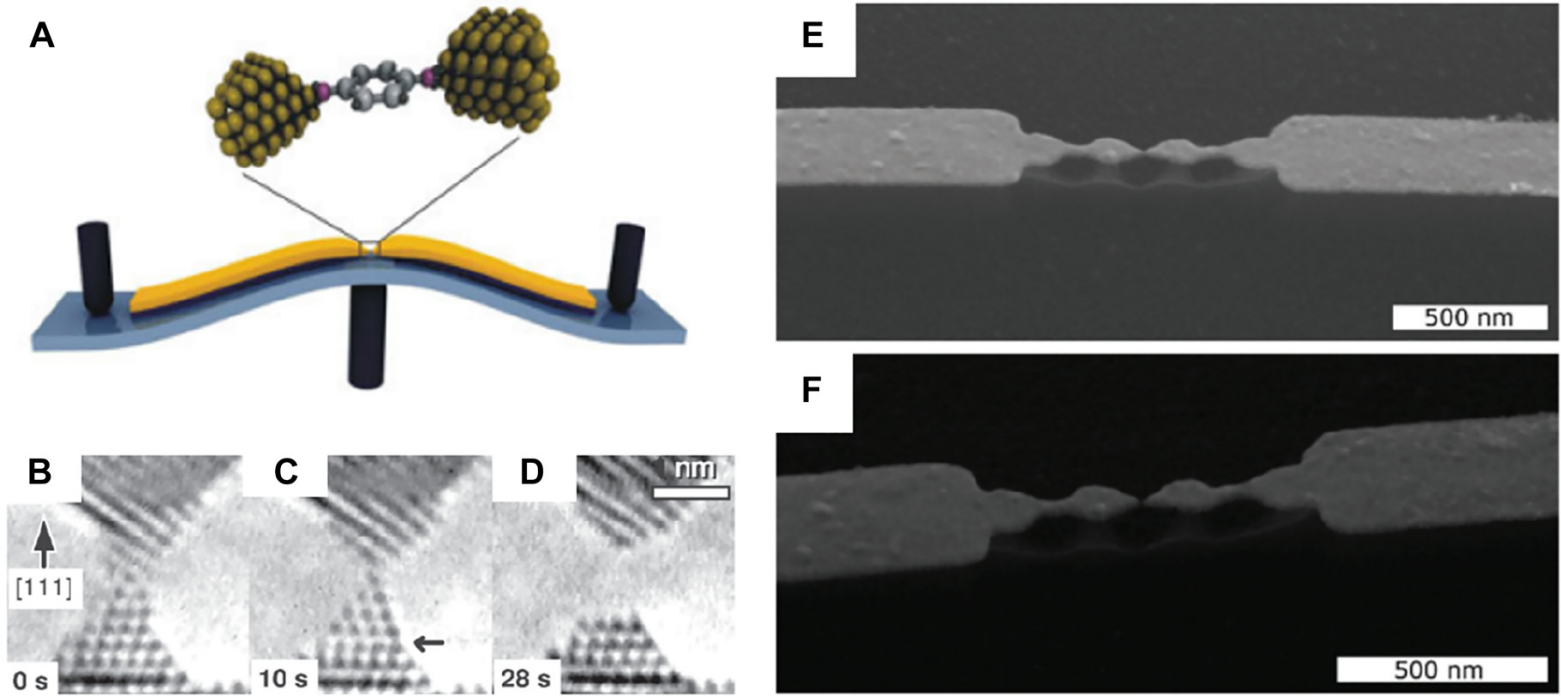
(A) Mechanically controllable break junction, commonly used for molecular electronic studies. (B)–(D) *in-situ* TEM images of an atomically thin metallic contact forming a break junction. (E) and (F) SEM images of a break junction before and after formation of the gap. This junction can support localized surface plasmon resonance and incorporate field enhancement at the gap. Reproduced with permission from [155] (A) [156], (B)–(D) [157], (E) and (F).

The most straightforward way of creating break junctions is to bend the whole structure to elongate the thinned down metallic wire and is commonly referred to as mechanically controllable break junctions (MCBJs), as depicted in Figure 14 [155]. The metallic wire is placed on top of a flexible substrate, which is normally fixed at both ends. Then a pushing rod, controlled by piezoelectric actuation, bends the beam to stretch the metallic wire and finally break the weak spot. In a study using time-resolved high resolution transmission electron microscope, snapshots of MCBJ formation in gold and platinum nanowires are obtained and the narrowest part of the wires are found to remain crystalline and defect-free until just before the breakage [156]. The ultraclean metallic contacts are very useful for making molecular junctions, but also can be used for optical applications. Laible et al. fabricated MCBJ-based bowtie antenna where the gap width can be monitored by measuring the current flowing through the junction and can be controlled with sub-angstrom precision [157]. The antenna also supports localized surface plasmon resonances at near-infrared, such that 785 nm irradiation leads to a strong electromagnetic field enhancement at the gap and consequent photon-induced tunneling. The structure may therefore be useful in realizing optoelectronic devices working in nanometer and subnanometer gap regime. Similar geometry has been utilized in studies where electronic transport and SERS of a single molecule was explored simultaneously [158, 159].

**Figure 15:**
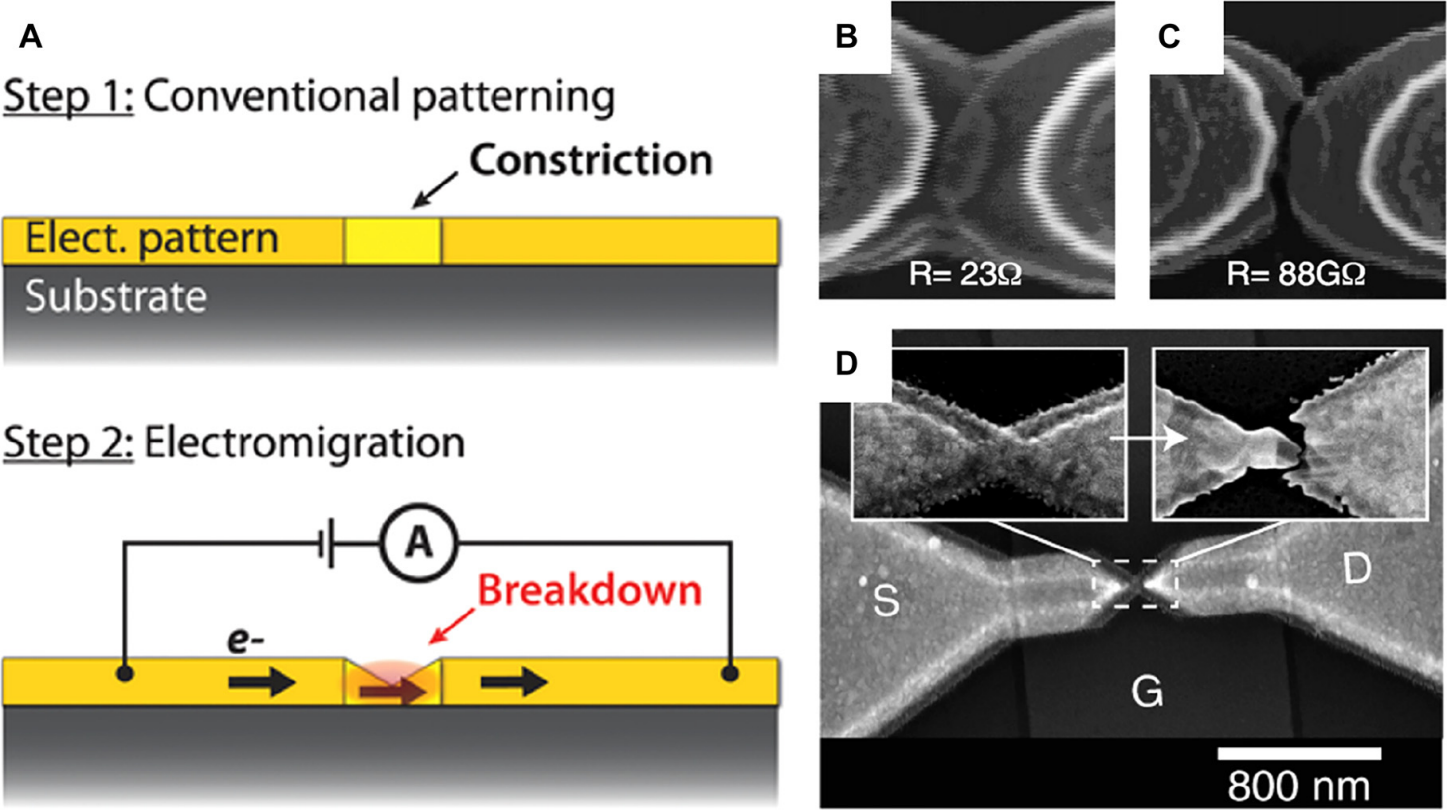
Fabricating nanometer-wide gaps with electromigration. (A) When electric current flows through a metallic pattern with a constriction, atoms diffuse gradually from the constriction, and eventually breakdown occurs to form a gap. (B) and (C) SEM images and resistance measured through the junction before and after electromigration process. (D) SEM image of a bowtie antenna fabricated with electromigration technique, operating at terahertz frequencies. Reproduced with permission from [160] (A) [161], (B) and (C) [29], (D).

Another well-established way of creating a gap from ‘zero-nanometer’ is electromigration-based break junctions, shown in Figure 15 [161]. Flow of large current through a thin metallic wire leads to electromigration of metallic atoms and eventually breaks the wire. Despite generally considered a failure in electronic circuitry, electromigration can be utilized to fabricate a few to one nanometer gaps in very large scale. Reliability of the process may be improved by controlling the temperature and current via feedback loops [162], [163], [164]. Similar to MCBJs, this method is generally used for electronic applications [165], [166], [167], [168], but it is also potentially useful for fabricating optical devices as well. In 2010, Ward et al. used this method to create plasmonic nanogap structure that incorporates a giant electric field enhancement and observe optical rectification as well as SERS enhancement [169]. The giant field enhancement at the gap may expand the structure via plasmonic heating, which enables switching of metallic point contacts by controlling the light intensity or polarization [170]. The tunnel junction may also be used as an efficient electroluminescence source as the local density of optical states is greatly enhanced at the subnanometer plasmonic gap [171]. Du et al. utilized the method to fabricate a bowtie antenna operating at terahertz frequencies with subnanometer antenna separation [29]. Due to extreme electromagnetic field confinement and enhancement at the gap, the authors were able to perform terahertz spectroscopy on a single fullerene molecule placed at the gap which is approximately 400,000 times smaller than the wavelength.

### 2.4 In-plane-oriented extended gaps: cracks

Breakjunctions described above are useful for creating point gaps but are not well suited for fabricating extended gap structures such as slits or slot antennas. Such extended gaps are unique especially for long wavelengths because incident light can only transmit through the gap, such that there exists a linear relationship between far field transmittance and near field enhancement at the gap [34, 172]. Accordingly, various quantitative studies on light–matter interactions including detection of molecules and biological systems [109, 173], [174], [175], carrier dynamics and phase transition in semiconductors [176], [177], [178], [179], high contrast optical switching devices [111, 180], etc. have been performed using these types of gap structures. It is therefore expected to be beneficial to realize extended metallic gaps that start from ‘zero-nanometer’ limit as well, and there is a well-known example – cracks.

**Figure 16:**
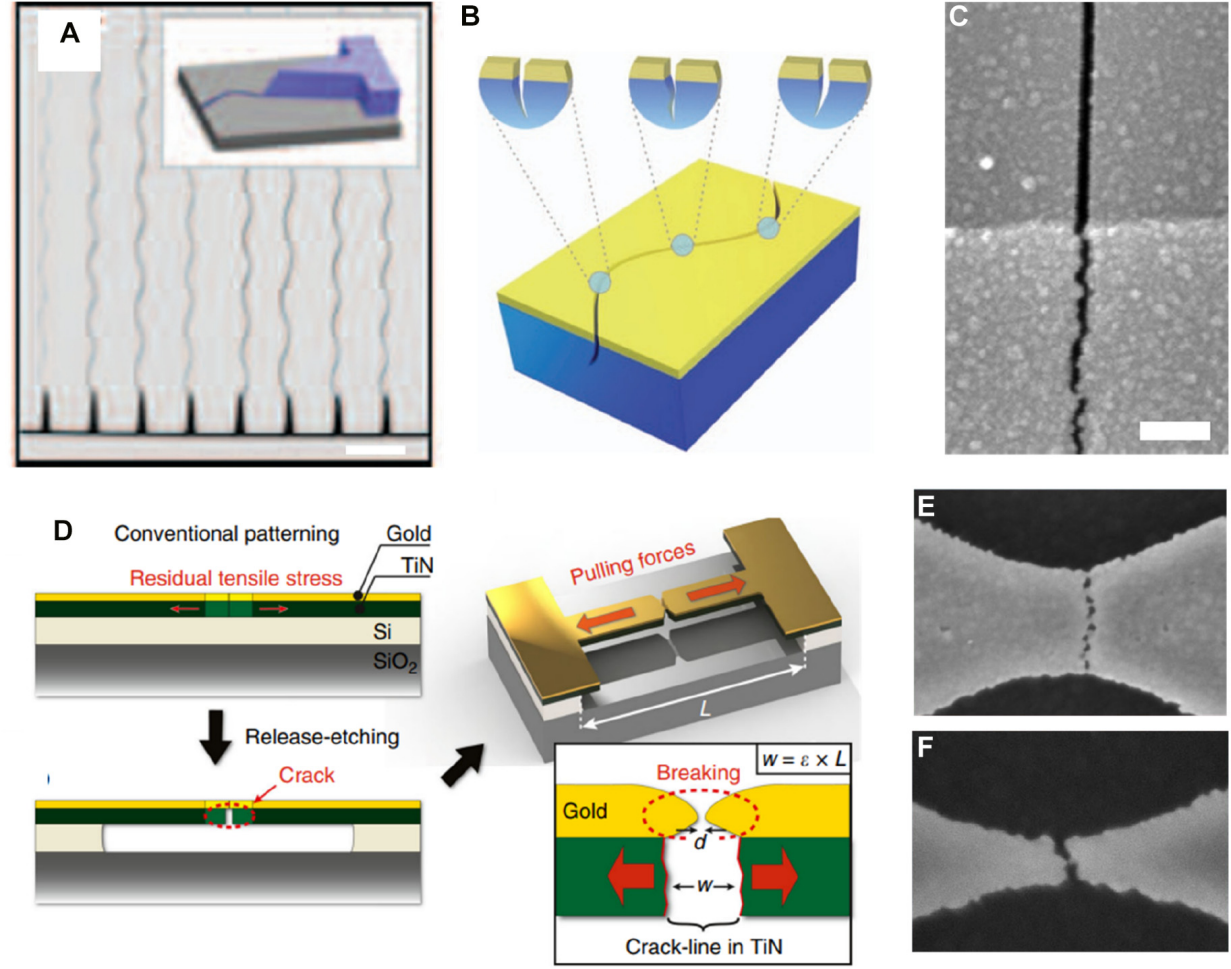
Nanopatterning with controlled cracking. (A) Patterning of an array of oscillating slits with cracks, initiated by a notch structure. (B) Formation of cracks by strain-controlled silicon nitride films deposited on a silicon substrate. (C) Metallic nanogaps fabricated on top of a silicon nitride film. (D) Schematic description of patterning parallel metallic gaps with pre-embedded crack lines in titanium nitride films. (E) and (F) Created metallic gaps with different bar widths. Scale bar: 100 nm (C). Reproduced with permission from [28] (A) and (B) [181], (C) [182], (D)–(F).

Cracks usually occur due to catastrophic release of stress in a thin film and tend to be formed randomly over the whole surface [183]. Despite such randomness, cracks have found unique applications in several electronic devices such as strain sensors [184], [185], [186], [187], [188]. For optical applications, however, the randomness must be suppressed for the created gaps to support resonances at desired wavelengths. Nam et al. demonstrated patternable nanogaps by controlled cracking in a strain-controlled silicon nitride film [28], and readily expanded the scheme to a thin metal film coated above (Figure 16A–C) [181]. The authors fabricated ∼10 nm-wide slit extending over several millimeters on a 15 nm titanium film with this method. In more recent research by Dubois et al. the authors embedded a pre-stressed titanium nitride bridge underneath a gold pattern and locally induced crack by release-etching the bridge structure [182]. The resulting gap width was sub-3 nm, and the method could be applied in a wafer-level. While gaps in these methods started from ‘zero-nanometer’, however, they cannot go back to the ‘zero-nanometer’ limit – that is, the cracks are irreversible and minimum achievable gap width is still limited to several nanometers.

**Figure 17:**
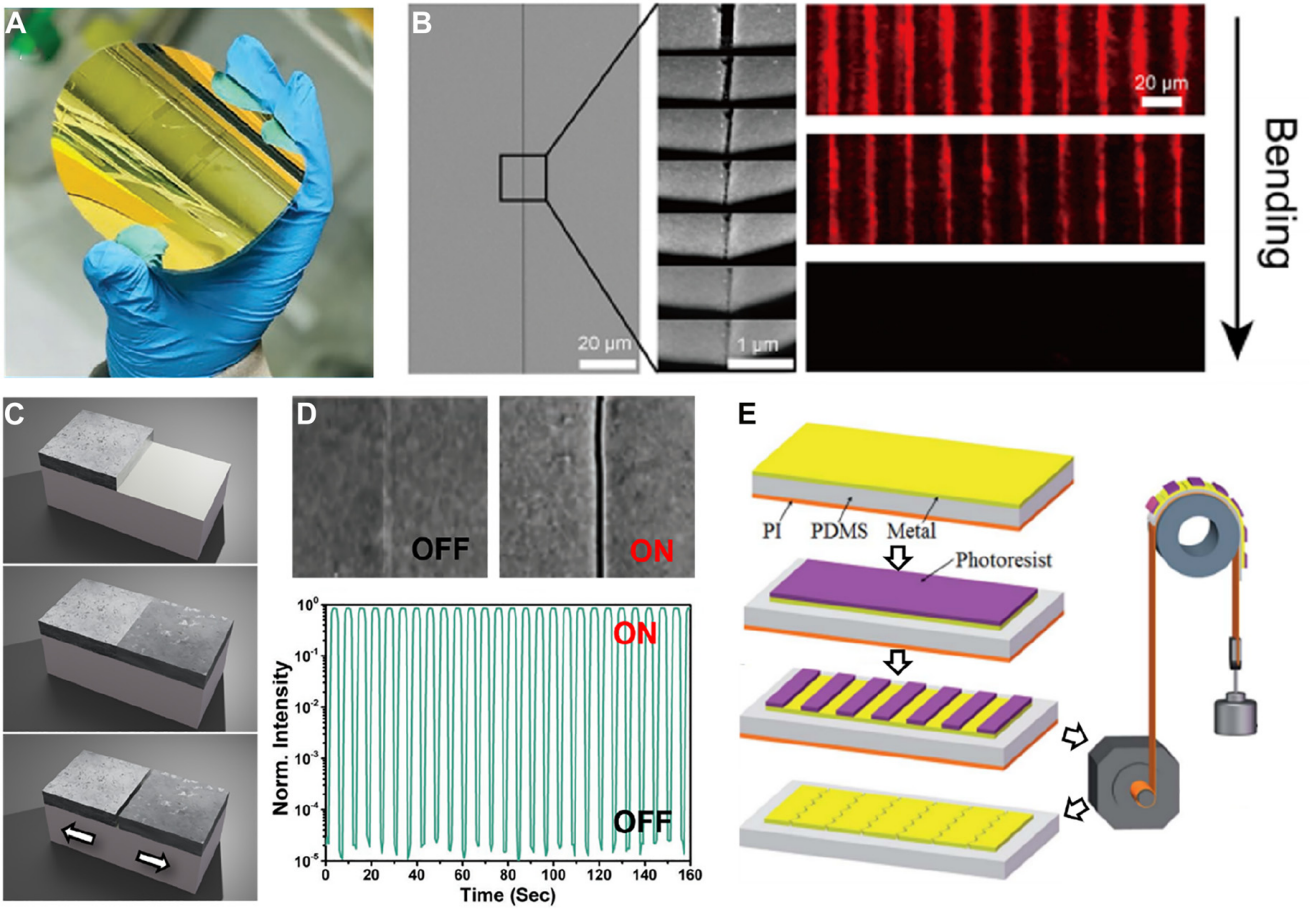
Healable crack-based metallic gaps fabricated on flexible substrates. (A) An optical image of wafer-scale metallic gaps on a flexible substrate. (B) SEM images and optical transmission images of metallic gaps upon bending the substrate and thereby closing the gap. (C) Schematic description of a ‘zerogap’, where a crack is prepatterned at the boundary of two metallic layers deposited at different times with different conditions. (D) SEM images of a ‘zerogap’ at ‘off’ and ‘on’ states, and transmittance through the gap array at relative states. (E) Patterning cracks on a PDMS substrate by photolithography and precisely controlled bending of the substrate. Reproduced with permission from [27] (A), (C) and (D) [189], (B) [190], (E).

One possible solution to overcome the abovementioned limit is to fabricate metallic gaps on a flexible substrate and shrink the whole structure to lessen the gap width, or ‘heal’ the gap. Kim et al. followed the approach by fabricating spacer-based metallic gaps on a PET substrate and then wet etching the gap-filling dielectric, as described above in Section 2-1 [189]. As the method is based on atomic layer lithography, the ‘healable gap’ can be fabricated in wafer-scale with essentially no limitation in the overall size of the gap (Figure 17). Upon bending the whole sample, slits narrowed from 20 to zero nanometers and became optically equivalent to a bare metallic film. The method shows high switching contrast especially at terahertz and microwave frequencies because only partial contact between the two metallic layers forming the gap can completely screen the gap, as shown in Section 1.3.

Das et al. also used a similar approach but defined the crack position *without* deposition of a spacer, such that the gap starts from zero nanometer and slowly opens as strain is applied [27]. This becomes possible by depositing two layers of metal at different times with different conditions, such that the boundary of the two layers do not homogenize as they do under pristine condition. Therefore, the method is essentially to embed prepatterned cracks in a lithographically controlled manner, as the authors call their sample ‘zerogap.’ Strength of this approach is that ‘off state’ – a state with no strain applied – corresponds to the smallest gap width, such that the structure is robust against fatigue or other sources of degradation. The slit array fabricated by the authors shows an on-off ratio of 10^5^ with transmission at ‘on state’ larger than 85% and show no fatigue after 10,000 times of switching operation.

Liu et al. also showed patterning of nanocracks, assisted by photolithography [190]. In this work, photoresist is first patterned on a thin metal film deposited on top of a polydimethylsiloxane/polyimide (PDMS/PI) substrate. Carefully bending the whole sample leads to selective formation of cracks at area without photoresist on top, because the strain of the exposed film becomes much larger than that of unexposed film during the bending process. By removing the resist, an array of closely packed (200 cm^−1^), 5 mm-long metallic slits are realized. This ‘pattern-and-pool’ method may be repeated multiple times to create additional gaps oriented in different directions. It is worth mentioning that such substrate tuning is not restricted to flexible elastomers; electromechanical modulation of semiconductor thin films can also be used for gap width tuning, and plasmonic dimers with separation of ∼0.9 nm have been realized as well [191].

## 3 Applications of nano- and zero-gaps

In previous chapters we made an overview on electromagnetic description of sub-10 to zero nanometer gaps and various means to fabricate them. The great variation of gap geometries and emerging phenomena enables a plethora of applications. While the gaps are by themselves already useful as photonic elements, integrating the gap with various semiconductors, molecules and quantum materials can also reveal pathways to many unexplored phenomena. In the following sections we will provide an overview of applications of nano- and zero-nanometer gaps. As nanophotonic sensing is very well understood and is described in detail in prior publications [95, 173, 192, 193] we would like to focus on some other aspects.

### 3.1 Strong coupling of light and matter

**Figure 18:**
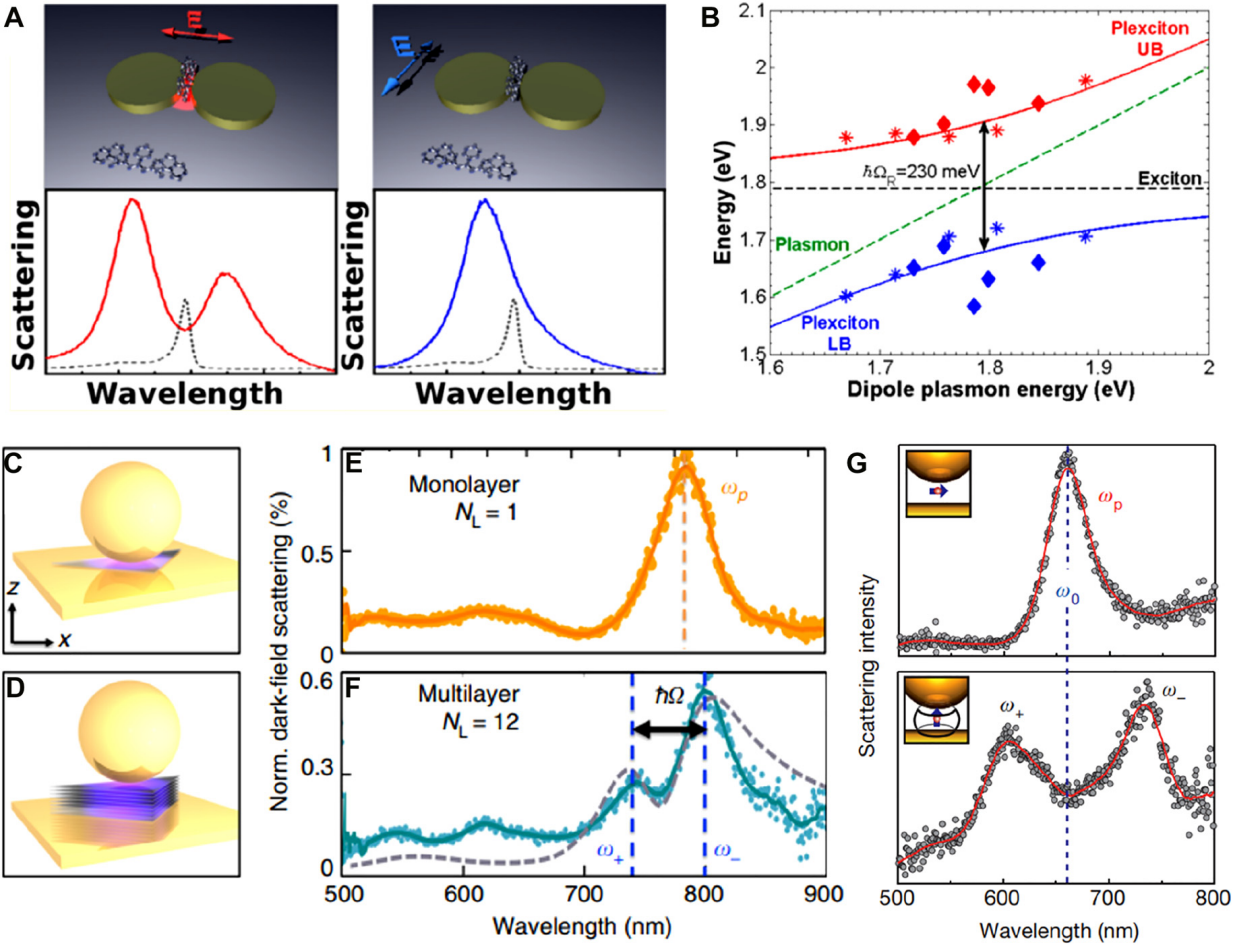
Strong coupling and Rabi splitting in nanogaps. (A) and (B) Strong coupling between plasmons in metallic nanodimers and excitons in J-aggregates leading to a formation of plexcitons and Rabi splitting of 230 meV. (C)–(F) Nanoparticle-on-a-mirror geometry with monolayer (C) and multilayer (D) WSe_2_ exhibiting no coupling (E) and strong coupling (F), respectively. G Strong coupling of dye molecules with gap plasmon mode, occurring only when the transition dipole moment of the dye molecules aligns with the enhanced electric field at the gap. Reproduced with permission from [211] (A) and (B) [30], (C)–(F) [212], (G).

One of the most important applications in nanophotonics is Purcell enhancement and consequent enhancement of light–matter interactions. While metallic nanogaps, due to Ohmic losses, generally suffer from relatively low quality factor of resonances compared to that of dielectric nanoresonators, they provide superior electromagnetic confinement and much smaller mode volume. Accordingly, many studies reported strongly enhanced light–matter interactions in plasmonic nanogap structures. When rate of energy exchange between material and photonic resonances is larger than any other energy loss rate in the system, the two resonances are said to be strongly coupled. At this regime the material and photonic modes hybridize and form polaritons and exhibit characteristic anticrossing behavior known as Rabi splitting [194]. Such strongly coupled systems may incorporate many interesting quantum phenomena including Bose–Einstein condensate [195], [196], [197], [198], ultralow threshold laser [199], [200], [201], [202], and single photon nonlinearity [203, 204], etc. The coupling strength is determined by transition dipole moment of the resonant material, local field enhancement introduced by the photonic mode and their spatial overlap. It is worth mentioning that it is possible to increase the transition dipole moment by means of collective coupling [205], [206], [207]. Also, in the density saturation limit where the optical modes are sustained solely by the materials themselves [208, 209], the coupling strength may reach an ultimate value regardless of the mode volume [210]. Nevertheless, in this section we focus on strong coupling phenomena observed in nanoplasmonic systems which can integrate seamlessly with various nanometer-sized and low-dimensional materials (Figure 18).

Schlather et al. observed Rabi splitting in a plasmonic nanodimer with 15 nm-wide gap coupled to J-aggregate molecules [211]. Here, bright mode of the plasmonic resonance strongly couples to excitonic resonance and exhibit Rabi splitting of 230 meV, which is 12% of the resonance frequency. The large Rabi splitting is attributed to a large transition dipole moment of the well-aligned J-aggregate complex. Kleemann et al. utilized nanoparticle-in-a-mirror geometry to achieve strong coupling in tungsten diselenide (WSe_2_) monolayer and multilayers [30]. In this work the authors find that strong coupling is not observed for monolayer WSe_2_ due to direction mismatch between in-plane excitons and out-of-plane field enhancement at the gap. When number of layers reaches seven or more, out-of-plane component of exciton arises due to multilayer-induced mixing and strong coupling with Rabi splitting of 140 meV is observed. The importance of alignment between enhanced electric field and transition dipole moment is also emphasized in research by Chikkaraddy et al., where the authors observe strong coupling in a 0.9 nm-thick film of methylene-blue dye only then the transition dipole moment is aligned parallel to the enhanced electric field at the gap [212]. Similar geometry was used by Park et al. for tip-enhanced strong coupling in a single quantum dot [213], and Ojambati et al. to demonstrate strong coupling of a plasmonic nanocavity with a single molecule and observe single photon emission [214].

### 3.2 Photochemistry

Photochemistry is another field that can take advantage of the huge field enhancement and charge transfer occurring at a few-, sub-, and zero-nanometer gaps. For reactions of stable chemical species, external energies corresponding to Gibb’s free energy differences between reactants and products, and/or the activation energy, are needed. While high temperature and pressure are generally required to provide the energy, photochemistry can take place in ambient conditions with the help of external structures. For instance, Honda et al. reported photodissociation of water into hydrogen and oxygen with TiO_2_ catalysis [215, 216], and most recently, photoreactions facilitating semiconductor and metallic nanostructures to produce H_2_ and to reduce CO_2_ are reported as well [217, 218]. These methods are normally based on transfer of photoexcited electron–hole pair of the external materials to the chemical reactant [219, 220]. Therefore, efforts have mostly been made to develop efficient photocatalysts that have optimal bandgap and to provide separated electrode positions for reduction and oxidation of chemical reactants [221], [222], [223].

Surface plasmonic structures can provide an additional pathway to enhance the activity of photocatalysts [224], [225], [226]. Photoexcited surface plasmon polariton modes not only help to localize the electromagnetic field on the surface of catalytic materials, but they also provide a pathway to transfer hot electrons over the Schottky barrier to neighboring photocatalytic semiconducting materials [225]. For example, TiO_2_ nanoparticles and electrodes with wide bandgap (∼3.3 eV), which normally can only be photo-excited with UV light, becomes susceptible to visible light when combined with plasmonic nanostructures [227]. Also, plasmonic nanostructures have been shown to enhance of photoreactions that form [228] and dissociate [229] chemical bonds of certain species.

**Figure 19:**
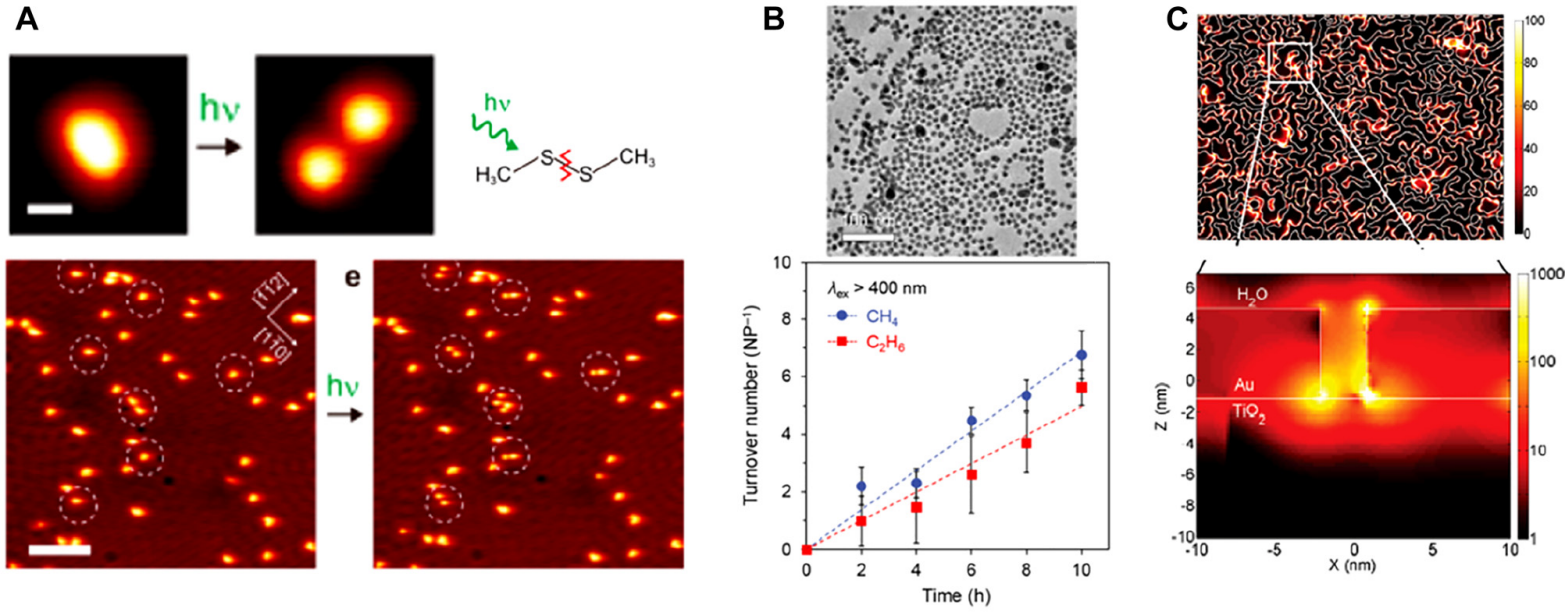
Experimental demonstration of plasmonic induced photoreaction. (A) STM monitored photo-dissociation of sulfide bonds of dimethyl disulfide on Ag or Cu surface with 532 nm excitation [237]. (B) Photo-induced CO_2_ conversion to CH_4_ and C_2_H_6_ via plasmonic excitation of Au nanoparticles [236]. (C) Plasmonic enhanced water splitting in Au nanogap structures [235].

Recently, photoreaction with only plasmonic metallic nanostructures has been demonstrated without using other catalytic materials. Considering the ultrashort lifetime of surface plasmon polariton (<100 fs) [230] which is mainly attributed to the electron-electron scattering process, photoreaction induced by only surface plasmon is expected to be marginal [231]. In such context, metallic gaps at zero-nanometer limit can provide an ideal testbed for efficient photoreactions, enabling either or both of the electric field localization and the electron tunneling between the gap [232]. For example, by placing metallic tip probe on a Ag surface coated with nitrothiophenol monolayer, dimerization process with excitation of 532 nm has been monitored in real-time [233]. The active control of photo-induced dimerization process was feasible with site-selectivity showing the merit of tip based geometry for photoreaction application. Also, photo-dissociation processes of the S–S bond of dimethyl disulfide on Ag or Cu surface have been observed by STM based gap in real-time [234]. In these reports, the light source lacked energy to excite the reactant molecules from highest-occupied to lowest-unoccupied molecular orbital states; the plasmonic enhancement of light at the gap helped overcome the barrier. Similar investigations to reduce or produce CO_2_ and H_2_ via plasmon-enhanced photoreaction were also reported [235, 236]. At sub- to zero-nanometer limit, plasmonic gap structures will provide much more extreme localization and enhancement of light, while providing an additional knob for hot electrons via charge transfer plasmon modes. Therefore, application of zero-nanometer gaps in photochemistry is promising in the aspects of environment friendly and economic CO_2_ reduction and H_2_ production (Figure 19).

### 3.3 Full control of long wavelength radiations for 5/6G communications

As described in Chapter 1 and 2, very small perturbation to an extended ‘zero-nanometer gap’ structure can lead to either full opening or complete closing of the gap, which means that complete switching of incident radiation is achievable with ultrasmall power consumption. When there are multiple gaps with different orientations, multiple functionalities can be added to the structure for control of amplitude, phase, and polarization of long wavelength radiation. Also, strongly enhanced light–matter interactions in nanogaps may enable nonlinear mixing of long wavelength radiation with visible or infrared light, which will greatly expand the applicability of terahertz communications.

**Figure 20:**
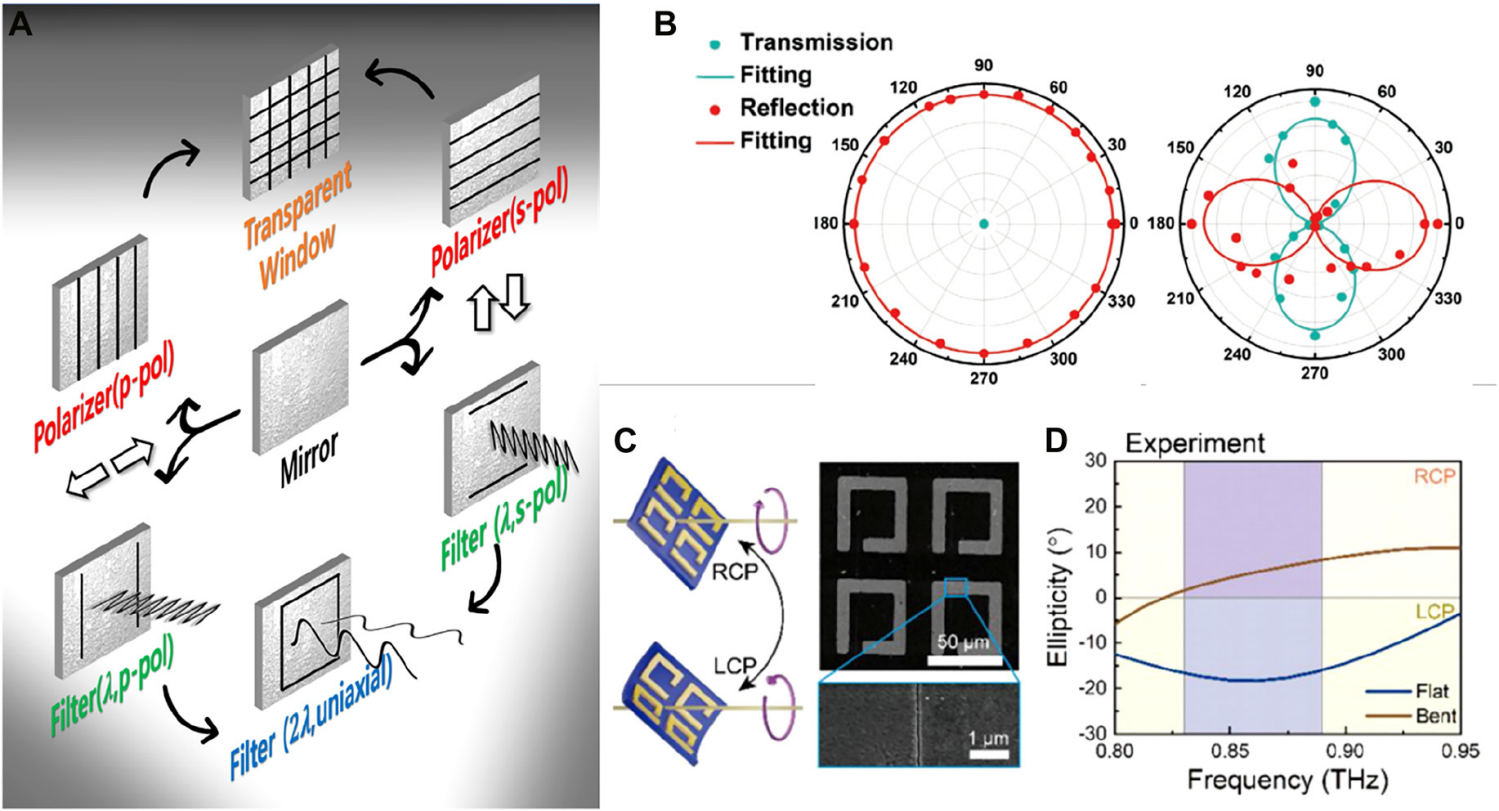
Full control of long wavelength radiations with zerogaps. (A) Various possible gap configurations and their respective optical functions. (B) Transformation from a mirror to a polarizer achieved in an array of closable slits. (C) Split ring resonators with embedded zerogaps exhibiting a change in topology upon opening and closing the gap. (D) Helicity switching with the topology-changing zerogaps. Reproduced with permission from [27] (B) [189], (C) and (D).

A simple yet powerful application is direct control of long wavelength radiations with extended ‘zero-nanometer gaps.’ Figure 20A demonstrate how ‘zero-nanometer gaps’ in the shape of a rectangular mesh or a coaxial aperture can transform into a polarizer, a filter or a mirror depending on the direction of strain applied. Also, with a more sophisticated design, the gap can perform as a waveplate and change helicity of incident light, as shown in Figure 20C. Therefore, the scheme of ‘zero-nanometer gap’ can be used to control every aspect of electromagnetic radiation – amplitude, phase, and polarization. Also, as it is expected to be possible to electrically actuate individual gaps in a programmable manner with an elastomeric substrate [238], the ‘zero-nanometer gaps’ may be embedded in the form of reconfigurable intelligent surfaces for re-routing and multiplexing wireless 6G communications [239]. It is also worth noting that such transformation occurs in much higher switching ratio and lower power consumption compared to other reconfigurable metasurfaces utilizing electro-mechanic actuation, optical excitation or phase change materials [240], [241], [242].

**Figure 21:**
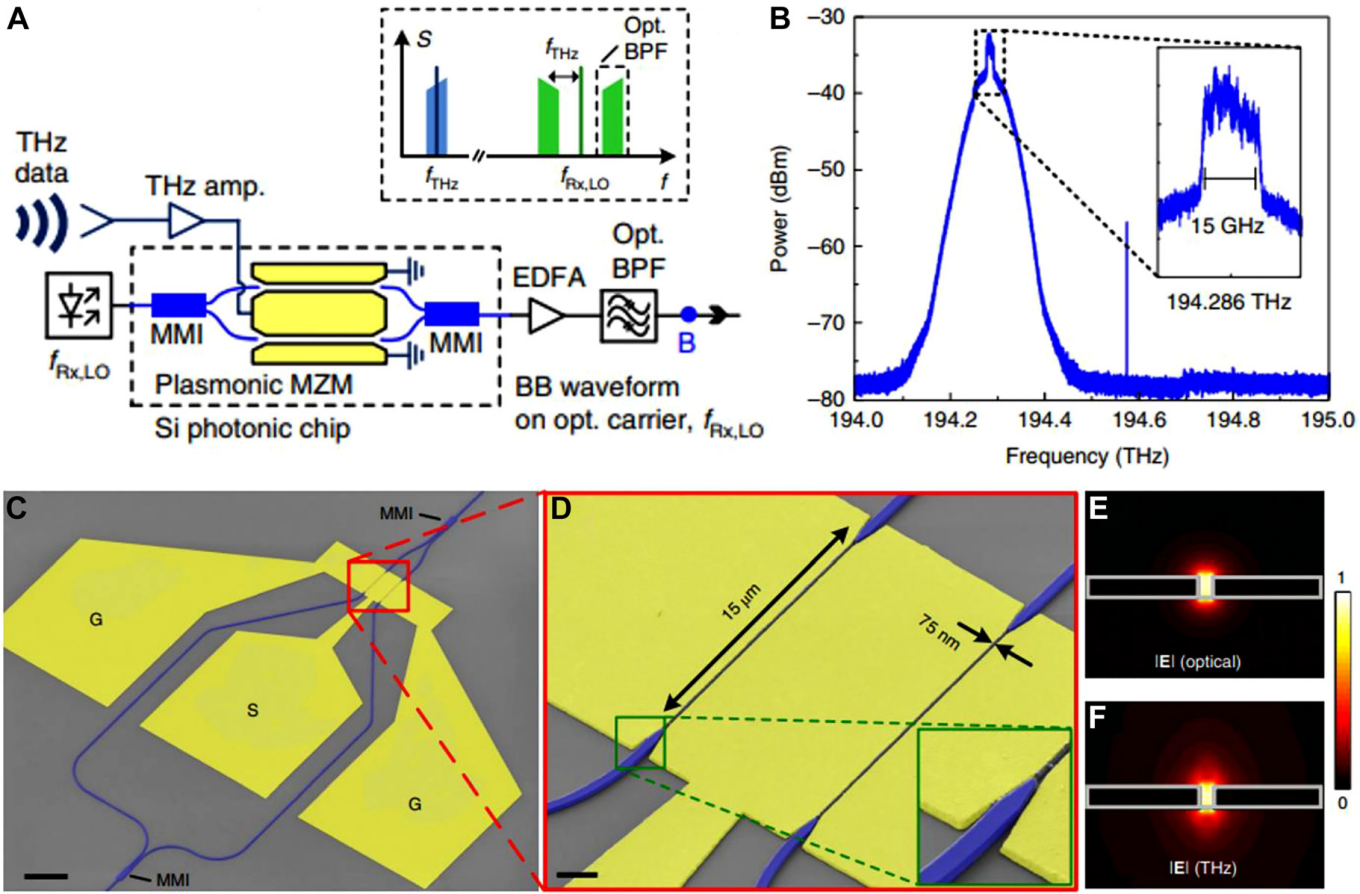
Nanogap-enabled THz-to-optical conversion of THz wireless signal to fiber-optic systems. (A) Concept of THz-to-optical conversion, where THz input is fed to a plasmonic Mach–Zehnder modulator and creates sidebands of an optical signal. Optical band pass filter is used to select one of the modulated sidebands. (B) Spectrum of a lower sideband, containing a 15 GBd quadrature phase-shifting keying (QPSK) signal centered at 194.286 THz. (C) and (D) False-colored SEM images of the plasmonic Mach–Zehnder modulator, comprising organic electro-optic material filled in a 75 nm-wide metallic slit. (E) and (F) Field profile of the optical and THz modes strongly confined to the gap. Reproduced with permission from [243].

Optoelectronic devices operating at millimeter and longer wavelengths are key to the future 5/6G communications and can benefit from the principles of nanophotonics as well. Ummethala et al. demonstrated THz-to-optical conversion in a plasmonic Mach–Zehnder modulator to integrate THz wireless links into a fiber-optic system [243] (Figure 21). The authors utilize a 75 nm-wide plasmonic slot waveguide which can incorporate both THz and optical field enhancement within the gap, and couple electro-optic (EO) materials to the gap. The enhanced THz field in the gap induces EO modulation of propagating optical beam and creates modulation sideband. Wireless data transfer over 16 m at a rate of 50 Gbit s-1 was demonstrated with the system. Key to the authors’ success is replacing a more commonly used EO material, lithium niobate, to an organic EO material which fits much easier into nanoplasmonic systems [244]. Overall performance and compactness of the device may be further improved by utilizing other low-dimensional, highly nonlinear materials such as transition metal dichalcogenides coupled to sub-10 nm plasmonic gap structures.

## 4 Discussions and outlook

In this review, we discussed various aspects of metallic nanogaps with emphases on underlying physics and applications in the limit of zero-nanometer gap width. As the gap width decreases to a few or sub-nanometer, gap plasmon effect and nonlocal effect start to appear. While zero-nanometer limit for point-like gaps is of interest for atomic imaging and local field enhancement, extended gaps operating at visible, far-infrared or longer wavelengths can be easily described by a simple circuit model combined with quantum conductance arising from conducting channels. It is these extended gaps that have enough volume to put to work in real world applications. In Chapter 2 we discussed various means to fabricate and control metallic gaps down to zero-nanometer limit. By utilizing cracks and combining them with flexible substrates one can control the gap size with picometer precision in wafer-scale, such that the gap can operate in technically all wavelengths spanning from visible, infrared to terahertz and even microwaves. Chapter 3 deals with possible applications of the metallic gap structures. While many applications utilize metallic gaps with a few-nanometer or much larger widths, their functionalities may be boosted by using subnanometer gaps and incorporating low-dimensional quantum materials as active layers. Especially, extended zero-nanometer gaps are expected to greatly benefit photonics at millimeter and longer wavelengths due to the unprecedented control over amplitude, phase, and polarization of the radiations that they have. With such ultrabroadband applicability and multilevel operation at subnanometer scale, dynamic metallic nanogaps can overcome the trivial role of providing electromagnetic field enhancement and may offer much more versatile platform for photonics under extreme electromagnetic environment, thereby opening the field of ‘gaptronics.’
